# Early Loss of Splenic Tfh Cells in SIV-Infected Rhesus Macaques

**DOI:** 10.1371/journal.ppat.1005287

**Published:** 2015-12-07

**Authors:** Félicien Moukambi, Henintsoa Rabezanahary, Vasco Rodrigues, Gina Racine, Lynda Robitaille, Bernard Krust, Guadalupe Andreani, Calayselvy Soundaramourty, Ricardo Silvestre, Mireille Laforge, Jérôme Estaquier

**Affiliations:** 1 Centre Hospitalier Universitaire (CHU) de Québec Research Center, Faculty of Medecine, Laval University, Québec, Québec, Canada; 2 CNRS FR3636, Faculty of Medecine des Saint-Pères, Paris Descartes University, Paris, France; 3 Life and Health Sciences Research Institute (ICVS), School of Health Sciences, University of Minho, Braga, Portugal; 4 ICVS/3B's-PT Government Associate Laboratory, Braga, Guimarães, Portugal; Emory University, UNITED STATES

## Abstract

Follicular T helper cells (Tfh), a subset of CD4 T lymphocytes, provide crucial help to B cells in the production of antigen-specific antibodies. Although several studies have analyzed the dynamics of Tfh cells in peripheral blood and lymph nodes (LNs) during Aids, none has yet addressed the impact of SIV infection on the dynamics of Tfh cells in the spleen, the primary organ of B cell activation. We show here a significant decrease in splenic Tfh cells in SIVmac251-infected rhesus macaques (RMs) during the acute phase of infection, which persists thereafter. This profound loss is associated with lack of sustained expression of the Tfh-defining transcription factors, Bcl-6 and c-Maf but with higher expression of the repressors KLF2 and Foxo1. In this context of Tfh abortive differentiation and loss, we found decreased percentages of memory B cell subsets and lower titers of SIV-specific IgG. We further demonstrate a drastic remodeling of the lymphoid architecture of the spleen and LNs, which disrupts the crucial cell-cell interactions necessary to maintain memory B cells and Tfh cells. Finally, our data demonstrated the early infection of Tfh cells. Paradoxically, the frequencies of SIV DNA were higher in splenic Tfh cells of RMs progressing more slowly suggesting sanctuaries for SIV in the spleen. Our findings provide important information regarding the impact of HIV/SIV infection on Tfh cells, and provide new clues for future vaccine strategies.

## Author Summary

Among CD4 T lymphocytes, follicular T helper cells (Tfh) are essential for B cell responses. Understanding the impact of viral infections on Tfh function, in particular in deep tissues such as the spleen, which is the main organ for B cell response, may be important for vaccine development. We used a non-human primate model of AIDS to study the effect of the viral infection on T and B cell subsets. In SIV-infected rhesus macaques, we demonstrated a depletion of splenic Tfh cells in the acute phase, together with a diminution of memory B cell frequencies. Moreover, we also showed that splenic Tfh cells harbor SIV DNA early after infection, which persists throughout SIV infection. Thus, splenic Tfh may represent a potential reservoir for HIV/SIV. Collectively, our data suggests that the loss of splenic Tfh cells, which sustain memory B cells, contributes to the lack of immune control against HIV/SIV infection.

## Introduction

The follicular T helper (Tfh) cell, part of the T helper cell populations, tightly controls germinal center (GC) development. Tfh are considered to be a distinct CD4 T cell type with great importance for protective immunity. Rare in the blood, Tfh are essential for maintaining GCs and mediate B cell affinity maturation [1,2]. Tfh provide survival and proliferation signals to B cells via multiple pathways, including CD40L, IL-21, and BAFF, which compete with Fas-FasL interactions [3–5]. IL-21 production by Tfh has an important function, as B cells are usually aberrant in the absence of IL-21 [4–6]. Moreover, IL-21 is a critical factor for the control of chronic viral infections [7–9]. Tfh cells selectively express CXCR5 and PD-1 [10,11] but only weakly CCR5, CCR2, CX3CR1, and related inflammatory cytokine receptors [12]. *BCL6* and *MAF* have been identified as master regulators of their differentiation [12–16].

It has been reported that during the acute and chronic phase of HIV infection, Tfh frequencies are increased in the blood [17], and especially in LNs of chronically-infected individuals [18]. Several groups, including ours, have shown that blood and LN Tfh cells are infected by HIV/SIV [18–24]. However, other groups have reported a loss of Tfh cells in blood [25]. HIV-infected individuals having less than 200 CD4 T cells/mm^3^ have a deficiency of IL-21-secreting CD4 T cells [26]. It has been proposed that Tfh cells isolated from peripheral LNs of infected individuals have a high spontaneous cell death rate [21], and limited proliferative capability [19]. Given the crucial role played by Tfh cells on B cell activation/differentiation, Cubas and colleagues have proposed that excessive and persistent triggering of PD-1 on LN Tfh cells may affect their ability to provide adequate B cell help [20]. In mice, even though PD-1 is associated with Tfh expansion in the spleen, it has been reported that in its absence Tfh are impaired in the synthesis of important cytokines for the differentiation of long-lived plasma B cells [27].

Recently, we demonstrated an abortive differentiation of splenic Tfh cells during the course of *Leishmania infantum* infection of rhesus macaques [28]. In this model, splenic Tfh cells fail to express PD-1 and are associated with abnormal B cell differentiation and poor production of parasite-specific IgG, despite the occurrence of polyclonal hypergammaglobulinemia [28]. These discrepancies may reflect organ-specific Tfh responses, yet no study on HIV/SIV infection has so far addressed the Tfh dynamics in the primary organ of B cell activation, the spleen. We hypothesized that the dynamics of splenic Tfh is affected during SIV infection and warranted investigation.

Herein, we show that splenic Tfh cells decrease early after SIV infection. This profound loss of Tfh cells is associated with the loss of memory B cell subsets and lower titers of SIV-specific IgG. Finally, whereas a transient increase in the transcriptional factors Bcl-6 and cMaf is observed, our results indicate an increase in the expression of the repressors of Tfh differentiation Foxo1 and KLF2. Due to the critical role played by Tfh cells in the establishment of the immune response, preventing both their depletion and infection could be a new challenge for vaccine strategies.

## Results

### Dynamics of lymphocytes is organ dependent

RMs of Indian origin were infected with SIVmac251 and sacrificed at different time points post infection to address the dynamics of CD4 and CD8 T cells, and B cells. Given that some MHC class-I alleles, particularly Mamu-A001, Mamu-B008 and Mamu-B017, are associated with low levels of viremia in Indian RMs [29–31], all the RMs used in this study were genotyped. In our cohort, only one homozygote for Mamu-B017 (PB055) was enrolled, and none of the other RMs resulted homozygote for the two other alleles (Table 1). At the chronic phase, a decrease in peripheral CD4 T cell counts was observed in all SIV-infected RMs except PB055. Viral load is also shown in the Table 1 at the death. At day>180, PB013 and PB044 showed lower levels of viral load compared to PB023 and PB028 (Table 1). The latter are sacrificed since they developed neurological and diarrhea disorders, and are identified in the manuscript as fast progressors, while PB013 and PB044 are identified as slow progressors [32].

**Table 1:** Genotypes of rhesus macaques included in our cohort. The table indicates the date of sacrifice, CD4 T cell loss (compared to the baseline), and viral load of uninfected (SIV-) and RMs infected with SIVmac251. Animals were genotyped for MHC class I *Mamu-A* and *Mamu-B* haplotypes.

	Animal OCID	Day of euthanasia post infection	ΔCD4 counts (Cells/mm3)	Viral load (copies/ml)	Mamu-A Haplotype 1	Mamu-A Haplotype 2	Mamu-B Haplotype 1	Mamu-B Haplotype 2
**SIV-**	PB057	0	323	0	A004	A006	B001a	B069a
	PB061	0	826	0	A006	A008	B001a	B024
	PB069	0	1350	0	A004	A012	B001a	B056a
	93750	0	ND	0	ND	ND	ND	ND
	528	0	ND	0	ND	ND	ND	ND
**SIV+**	PB038	14	ND	0	A006	A011	B001a	B001a
	PB052	14	898	7.90 x10^4^	A002	A008	B069a	B106
	PB006	14	74	2.81 x10^7^	A001	A023	B002	B056a
	PB041	14	-554	8.94 x10^6^	A025	A026	B008	B017f
	PB005	14	-784	5.81 x10^6^	A008	A008	B001a	B012a
	PB051	14	116	4.67 x10^7^	A004	A004	B012b	B012b
	PB049	30	493	9.12 x10^7^	A002	A004	B069a	B069a
	PB026	30	-947	2.34 x10^6^	A007	A008	B008	B008
	PB015	30	-492	1.11 x10^6^	A008	A224	B001a	B001a
	PB033	30	-160	6.54 x10^6^	A002	A008	B001a	B017a
	PB030	30	-954	2.08 x10^7^	A008	A008	B015a	B017a
	PB055	30	257	3.24 x10^7^	A008	A019	B017a	B017a
	PB023	> 180	-761	1.39 x10^8^	A001	A006	B001a	B015b
	PB028	> 180	-668	1.61 x10^8^	A004	A012	B012b	B043a
	PB044	> 180	-216	5.71 x10^7^	A008	A008	B008	B017a
	PB013	> 180	-1404	3.57 x10^6^	A001	A008	B002	B055

**Fig 1:**
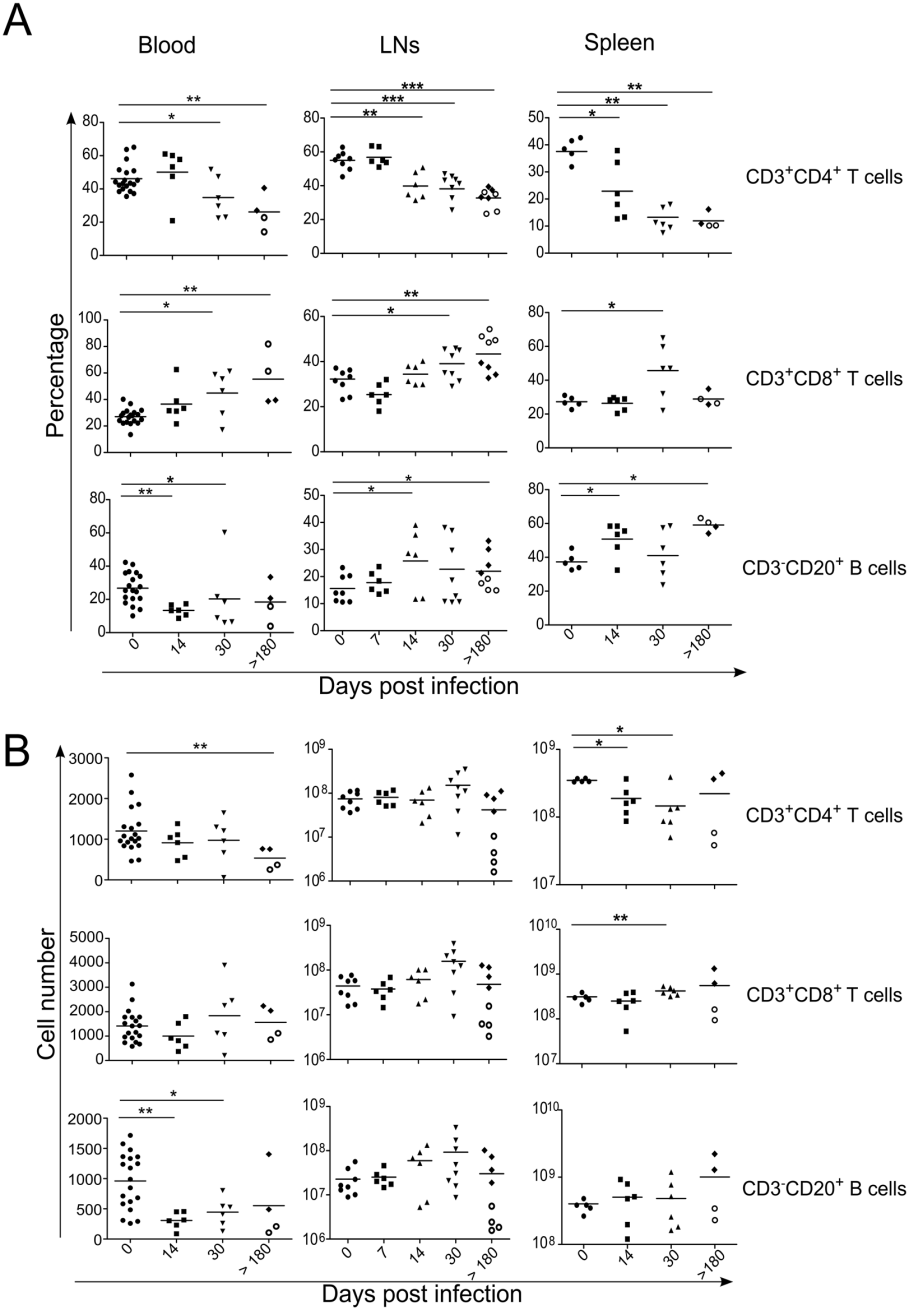
T and B cell dynamics in *SIV*-infected rhesus macaques. (A) Flow cytometry was used to determine the percentage of CD3^+^CD4^+^ and CD3^+^CD8^+^ T cells, and CD3^-^CD20^+^ B cells in the (Left panel) blood, (Middle panel) LNs and (Right panel) spleen. (B) Histograms show total number of T and B cells in LNs and the spleen, and cell number/mm^3^ in peripheral blood. Each dot represents an individual RM; Axillary and Inguinal LNs are represented separately by two distinct dots for each RMs at day>180. Statistical analyses were performed using Mann Whitney test. *, p<0.05; **, p<0.01. At day>180, open circles represent fast progressor RMs PB023 and PB028; and full diamonds represent slow progressor RMs, PB013 and PB044.

In order to study the dynamics of CD4, CD8 and CD20 cells, the samples were recovered in peripheral blood, LNs (axillary and inguinal LNs) and spleen, over the time course of infection. Results in peripheral blood showed, as expected, a significant decrease in the percentage of CD3^+^CD4^+^ T cells concomitantly with the increase in the percentages of CD3^+^CD8^+^ T cells (Fig 1A, left panel); the percentages of B cells (CD3^-^CD20^+^) were also diminished in SIV-infected RMs compared to healthy animals (day 0, 26.77% ± 9.3%; day 14, 13.35% ± 3.36%; *p* = 0.0013) (Fig 1A, left panel). The dynamics of total CD3^+^ T cells is shown in supplemental S1 Fig.

In peripheral LNs (additional peripheral LNs were recovered at day 7 to analyze earlier T and B cell dynamics), the percentage of CD3^+^CD4^+^ T cells decreased whereas the percentage of CD3^+^CD8^+^ T cells significantly increased at day 30 post-infection (CD3^+^CD4^+^ T cells, day 30, 39.76% ± 7.23%; day 0, 54.31% ± 5.95%; p = 0.0003 and CD3^+^CD8^+^ T cells, day 30, 38.9%± 6.93%; day 0, 30.20% ± 4.78%; p = 0.0141) (Fig 1A, middle panel). Total cell numbers were also evaluated. Only minor changes were observed in the LNs with the exception of the two animals having higher viral loads (PB023 and PB028, open circles) at the chronic phase (> 180 days). Thus, the lymphopenia observed in these animals resulted not only from CD4 T cell depletion but also CD8 T cells and B cells (Fig 1B, middle panel).

In the spleen, the decrease in the percentage of CD3^+^CD4^+^ T cells is related to an increase in the percentages of B cells and CD3^+^CD8^+^ T cells (Fig 1A, right panel). However, we clearly observed a progressive reduction in the numbers of CD3^+^CD4^+^ T cells early after the infection, which persists in the two animals with higher viral loads (PB023 and PB028). These two RMs displayed also lower numbers of splenic B cells and CD3^+^CD8^+^ T cells (Fig 1B, right panel) compared to the two slow progressors (PB013 and PB044); the latter having even higher B cell and CD8 T cell counts than that observed in healthy RMs.

Globally, our data revealed dynamic changes in the main lymphoid populations, which were organ dependent.

### Depletion of splenic memory Tfh CD4 T cells early after infection

**Fig 2:**
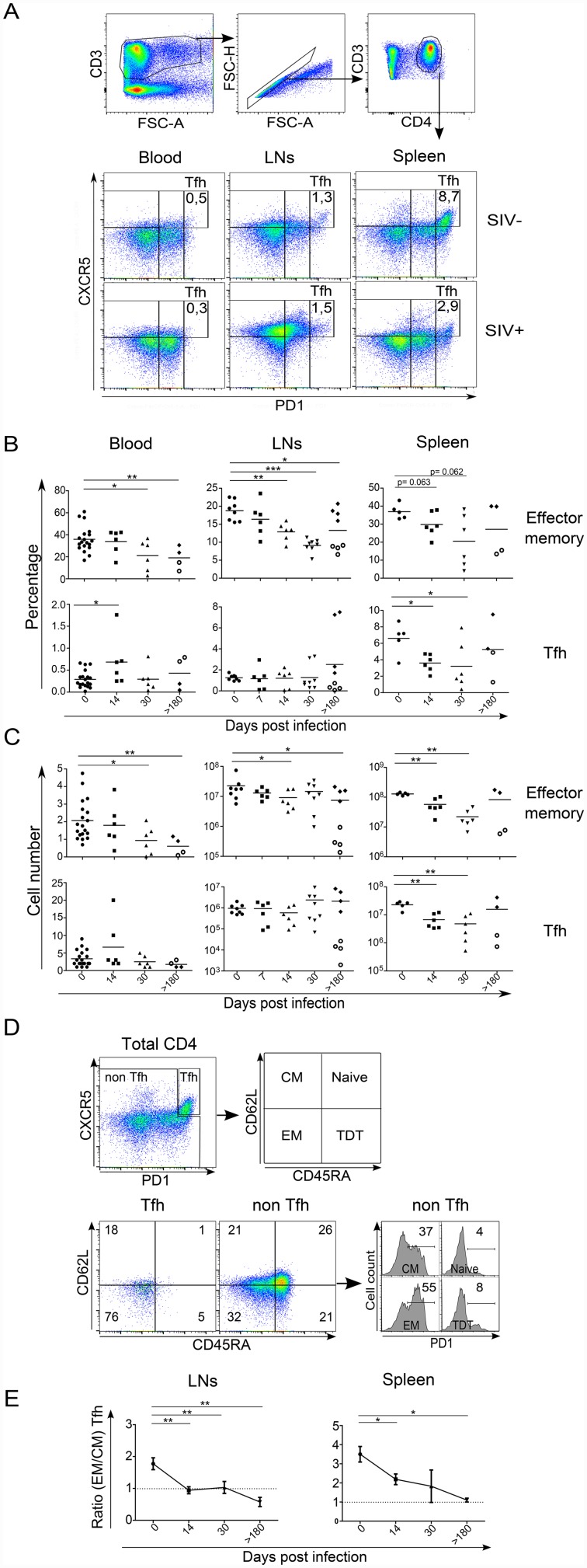
Early loss of splenic Tfh cells in *SIV*-infected rhesus macaques. (A) Representative dot plots depicting the expression of CXCR5 and PD-1 in the blood, in LNs and the spleen of either a non-infected RM (SIV^-^) or a SIV-infected RM at day 14. CD3^+^ T cells were first gated (CD3 against FSC-A), and then doublets were eliminated (FSC-H against FSC-A). Among the CD3^+^CD4^+^ T cell population, we assessed the expression of CXCR5 and PD-1. (B) Percentages and (C) cell numbers (expressed in cell number/mm^3^ in the blood) of effector memory (CD45RA^−^CD62L^−^) CD3^+^CD4^+^ T cells and Tfh (CXCR5^+^PD-1^bright^) cells are shown. Each dot represents an individual RM. Axillary and Inguinal LNs are represented separately by two distinct dots for each RMs at day >180. Statistical analyses were performed using Mann Whitney test. *, p<0.05; **, p<0.01. At day>180, open circles represent fast progressor RMs PB023 and PB028; and full diamonds represent slow progressor RMs PB013 and PB044. (D) Expression of CD45RA and CD62L on both splenocyte Tfh (CXCR5^+^PD-1^bright^) and non-Tfh cell subsets isolated from a non-infected RM; naive (CD45RA^+^CD62L^+^), central memory (CD45RA^−^CD62L^+^), effector memory (CD45RA^−^CD62L^-^) and terminally differentiated (CD45RA^+^CD62L^−^). The expression of PD-1 was also assessed on non-Tfh cell subsets. Numbers indicate the percentage of each subpopulation. (E) Histograms show the ratio of EM versus CM Tfh cells in LNs and spleen of RMs at the indicated time. Statistical analyses were performed using Mann Whitney test.

Depletion of memory CD4 T cells is a central event in the occurrence of AIDS [33–36]. Given that memory CD4 T cells also include Tfh, we analyzed the dynamics of these two subsets independently. Tfh cells were defined by the simultaneous expression of PD-1 and CXCR5 (CXCR5^+^PD-1^bright^) (Fig 2A). We assessed their dynamics in peripheral blood, LNs and in the spleen. Consistent with its role as the major organ for the initiation of humoral response, the percentage of Tfh cells in the spleen is at least five to ten fold higher than either in LNs or peripheral blood of healthy RMs (Fig 2A and 2B). Interestingly, we found that SIV infection is associated with a rapid decline in the percentage of splenic Tfh cells (day 0, 6.60% ± 1.88%; day 14, 3.61% ± 1.06%; *p* = 0.0152) (Fig 2B, right panel), and a transient increase in the blood (day 0; 0.29% ± 0.19%; day 14, 0.68% ± 0.56%; *p* = 0.040). Splenic Tfh cell numbers decreased about five fold at day 14 post-infection as compared with uninfected RMs (Fig 2C). In chronically SIV-infected RMs, the percentages and numbers of splenic Tfh in slow progressors (PB013 and PB044) were equivalent to those observed in healthy RMs, whereas both were drastically reduced in fast progressors (P023 and PB028) (Fig 2B and 2C). Although a trend toward a decrease in the percentages of effector memory CD4 T cells is observed at day 14 p.i., suggesting that such early depletion is specific to Tfh cells (Fig 2B, right panel), their numbers decreased indicating a more global effect of SIV infection on the pool of splenic memory CD4 T cells during the acute phase (Fig 2C, right panel). Mostly, the numbers of splenic central memory (CD45RA^-^CD62L^+^) and terminal differentiated (CD45RA^+^CD62L^-^) CD4 T cells decreased during the acute phase (S2 Fig).

Consistent with our observation that cell dynamics is organ dependent, we found minor changes in the percentages and numbers of Tfh cells in the LNs during the acute phase (Fig 2B and 2C, middle panel). The absence of Tfh depletion during the acute phase (day 14) contrasts with the depletion of effector memory CD4 T cells (Fig 2C). However, and consistent with previous reports indicating an increase in the percentages of Tfh cells in peripheral LN of chronically SIV-infected macaques [20,21,23], we found that the increase on the percentages of Tfh cells was restricted to slow progressor RMs displaying a lower viral load and higher CD4 T cell counts (PB013 and PB044) (Fig 2B, middle panel). Thus, fast progressors (PB023 and PB028) displayed LNs T cell depletion of both Tfh and effector memory CD4 T cells consistent with global lymphopenia of memory cells observed in those animals.

Therefore, we demonstrated an early and persistent depletion of splenic Tfh cells, particularly in fast progressor RMs.

### Abortive differentiation of memory Tfh cells reflects curtailed expression of Tfh-specific transcription factors

Because central memory are less prone to die than effector memory CD4 T cells of HIV- and SIV-infected individuals [33,36–40], distinct phenotype of Tfh may be a possible explanation in their differential dynamics in LNs and spleen. Therefore, we analyzed the expression of CD62L and CD45RA in fresh Tfh cells. Consistent with previous reports [6,41], splenic Tfh cells from healthy RMs were mostly effector memory cells (CD45RA^-^CD62L^-^) and less than 30% of them have a central memory phenotype (CD45RA^-^CD62L^+^; day 0, 22.11% ± 4,60%) (Fig 2D). The ratio of effector memory versus central memory Tfh in healthy RMs is 3.5 in the spleen and 1.77 in LNs (Fig 2E). Interestingly, whereas early after infection splenic Tfh cells from SIV-infected RMs maintain an effector memory phenotype, LNs Tfh cells switch to a central memory phenotype (Fig 2E). In chronically SIV-infected RMs, both in the spleen and LNs, the EM:CM ratio of the Tfh compartment is significantly lower as compared with pre-infection (Fig 2E). Thus, our results indicate the accumulation of central memory Tfh cells in SIV-infected RMs, particularly in LNs.

**Fig 3:**
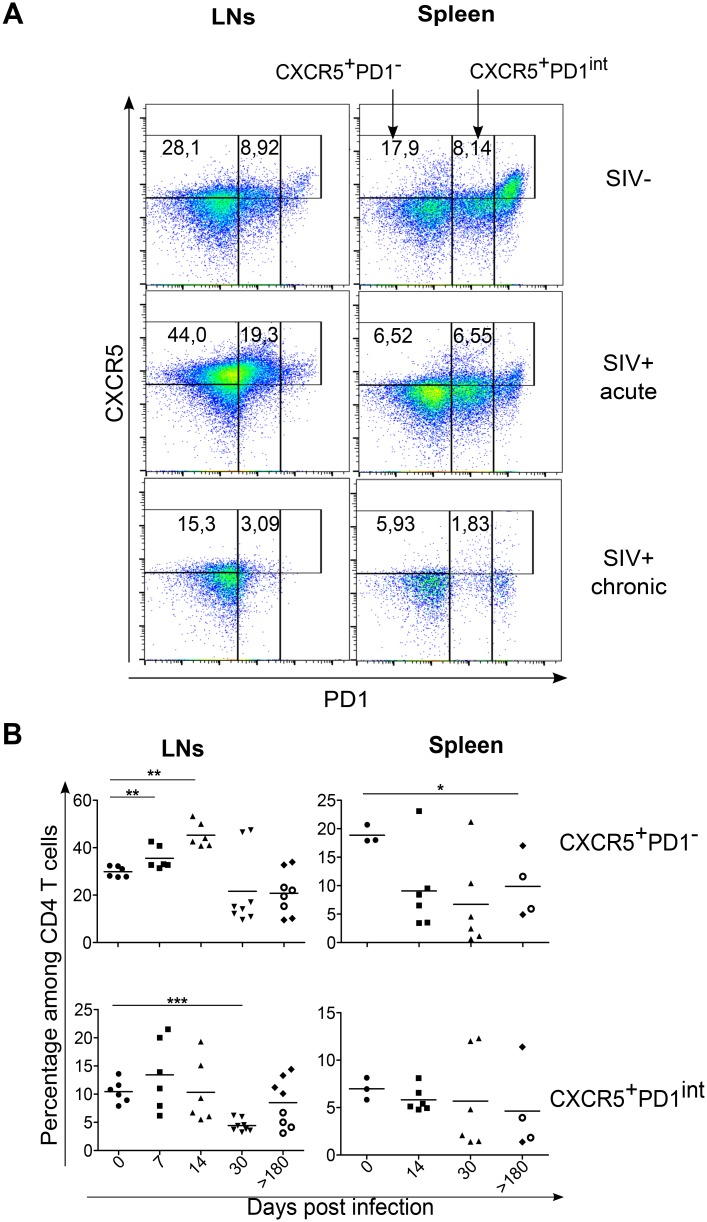
Transient increase of LNs CXCR5^+^CD4^+^ T cells during SIV infection. (A) Representative dot plots depicting the expression of CXCR5 and PD-1 among CD3^+^CD4^+^ T cells. The percentages of CXCR5^+^PD-1^int^ and CXCR5^+^PD-1^-^ are indicated in LNs and the spleen of either a non-infected RM (SIV^-^), a SIV-infected RM at day 14 (SIV^+^ acute), and a SIV-infected RM at day >180 (SIV^+^ chronic), (B) Histograms showing the percentages of both CXCR5^+^PD-1^int^ and CXCR5^+^PD-1^-^ among CD4 T cells in LNs and the spleen. Each dot represents an individual RM. Axillary and Inguinal LNs are represented separately by two distinct dots for each RMs at day>180. Statistical analyses were performed using Mann Whitney test. *, p<0.05; **, p<0.01. At day>180, open circles represent fast progressor RMs PB023 and PB028; and full diamonds represent slow progressor RMs PB013 and PB044.

Because we recently reported the transient expansion of a population of CXCR5^+^ Tfh-like cells that fail to express PD-1 during *L*. *infantum* infection of RMs [28], we analyzed more in detail the dynamics of CD4 T cells expressing CXCR5 and PD-1 during SIV infection (Fig 3A). In the LNs, early after infection, a significant transient increase of CXCR5^+^PD-1^-^ is observed in all infected RMs peaking at day 14 (CXCR5^+^PD-1^-^, 45.28% ± 5.17%; *p* = 0.0011) compared to healthy RMs (CXCR5^+^PD-1^-^, 29.85% ± 2.26%) (Fig 3B, left upper panel). However, by day 30 and thereafter, the percentages of both CXCR5^+^PD-1^-^ and CXCR5^+^PD-1^int^ subsets declined and reached values lower than those observed before infection, particularly in fast progressors (Fig 3B, left bottom panel). In the spleen as well as in the LNs, a trend towards a decrease was observed at day 30 (Fig 3B, right panel). Thus, in chronically SIV-infected RMs both in the spleen and LNs, loss of CXCR5^+^PD-1^-^ and CXCR5^+^PD-1^int^ subsets were observed that could be an indication of abortive differentiation.

**Fig 4:**
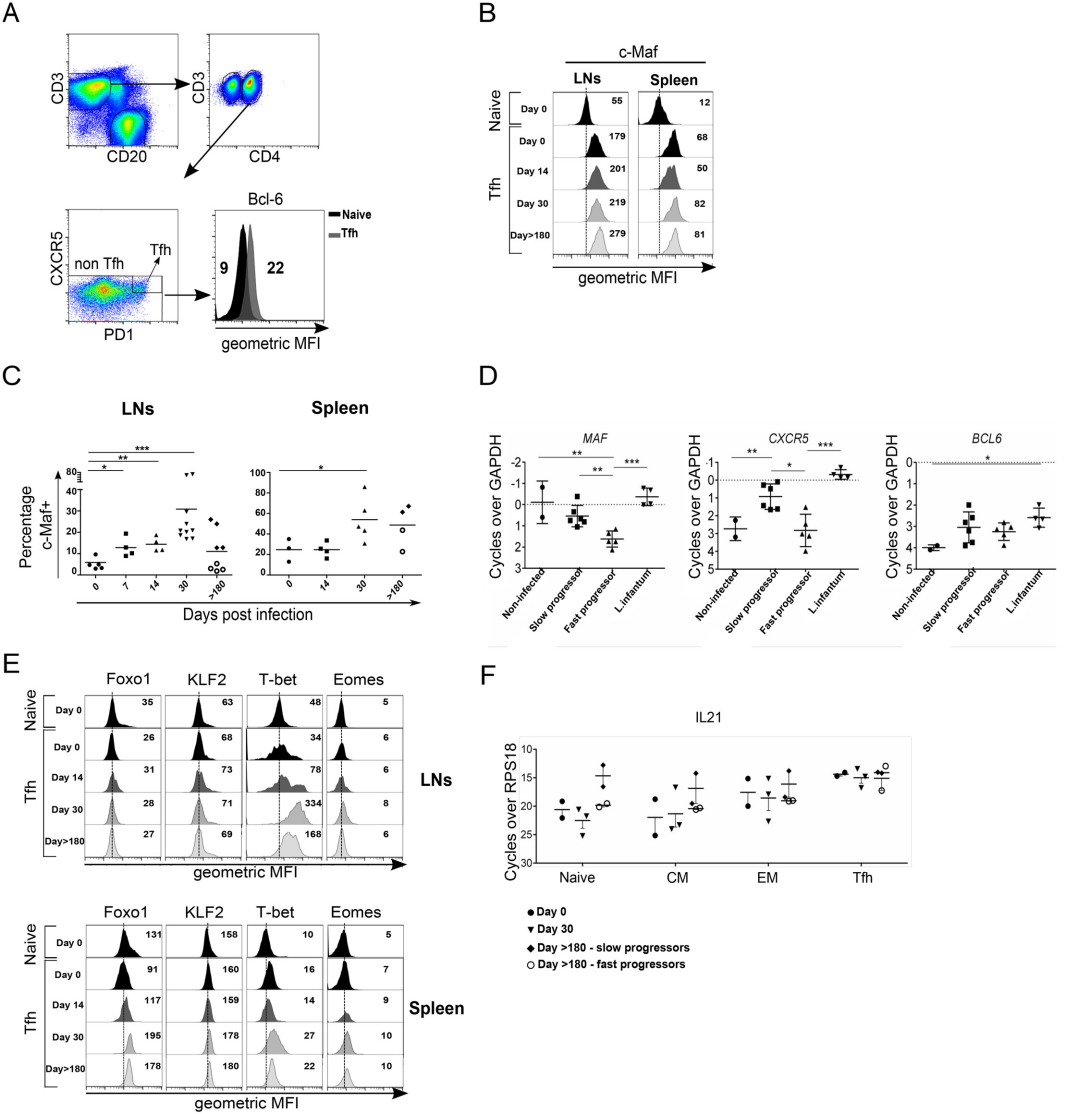
Changes in the expression of transcriptional factors associated with the differentiation of Tfh cells during SIV infection. (A) Representative expression of Bcl-6 in Tfh (CXCR5^+^PD-1^bright^) and naïve non Tfh CD3^+^CD4^+^ T cells in the spleen of a healthy RM. (B) Mean fluorescence intensity (geometric) of c-Maf expression in Tfh (CXCR5^+^PD-1^bright^) and naïve non Tfh CD3^+^CD4^+^ T cells in both the LNs and spleen of RMs at different days post-infection. (C) Percentages of c-Maf^+^ Tfh cells in LNs and spleen. Each dot represents an individual RM. Statistical analyses were performed using Mann Whitney test. *, p<0.05; **, p<0.01. At day>180, open circles represent fast progressor RMs PB023 and PB028; and full diamonds represent slow progressor RMs PB013 and PB044. Axillary and Inguinal LNs are represented separately by two distinct dots for each RMs at day>180. (D) Histograms show RT-PCR quantification of *BCL6*, *MAF* and *CXCR5* mRNA in splenocytes of chronically SIV-infected RMs of Chinese origin compared to *Leishmania infantum*-infected RMs, and healthy RMs. Each dot represents an individual RM. Statistical analyses were performed using Mann Whitney test. *, p<0.05; **, p<0.01. (E) Histograms show geometric mean fluorescence intensity (MFI) of Foxo1, KLF2, T-bet and Eomes in Tfh cells of RMs at the indicated time compared to naïve non Tfh CD3^+^CD4^+^ T cells of an healthy RM. LNs (upper panel) and spleen (bottom panel) are shown. (F) RT-PCR quantification of IL-21 mRNA expression in naive, central memory (CM), effector memory (EM), and Tfh cells of SIV-infected RMs.

Transcription factors such as Bcl-6 and c-Maf are essential for inducing and maintaining Tfh differentiation [42]. Consistent with the differentiation of Tfh cells, we found higher level of Bcl-6 in Tfh cells compared to naïve CD4 T cells (Fig 4A). Interestingly, c-Maf expression in Tfh cells increased both in LNs and spleen of SIV-infected RMs (Fig 4B). We found a significant transient increase in the percentage of c-Maf^+^ -expressing Tfh cells plateauing at day 30 post-infection in both LNs (30.83% ± 21.12%, *p* = 0.0001) and spleen (54.00% ± 21.21%; *p* = 0.035) compared to healthy RMs (LNs, 5.85% ± 3.02% and spleen, 24.68% ± 11.10%, respectively) (Fig 4C). These percentages then diminished at the chronic phase (day>180), but nevertheless remained higher in slow progressors (PB013 and PB044) compared to fast progressors (PB023 and PB028) and healthy animals (Fig 4C).

To determine whether splenocytes of chronically SIV-infected RMs of Chinese origin, similarly to Indian RM, displayed a difference in the expression of transcription factors, we quantified *BCL6*, *MAF* and *CXCR5* mRNA expression by RT-PCR. Because in Chinese monkeys we have previously reported distinct profile of progression [36,43,44], we analyzed their expression in fast and slow progressor RMs, and compared their expression to *L*. *infantum*-infected RMs as we recently reported [28,45]. Our results clearly indicate lower levels of *MAF* and *CXCR5* mRNA in fast progressor SIV-infected RMs compared to either slow progressor SIV-infected RMs or *L*. *infantum*-infected RMs (Fig 4D).

Recently, it was reported that expression of the Krüppel-like factor 2 (KLF2) restrains Tfh cell differentiation inhibiting CXCR5 and Bcl-6 expression [46,47]. Moreover, KLF2 is one the genes targeted by Foxo1, which has been also shown to negatively regulate the differentiation of Tfh cells through at least the involvement of the E3 ubiquitin ligase Itch [48,49]. KLF2 as well as Foxo1 were reported to regulate the expression of CD62L [46,50,51]. Thus, we examined the expression of Foxo1 and KLF2 in Tfh cells by flow cytometry (Fig 4E). Whereas the expressions of KLF2 and Foxo1 remained mostly unchanged in Tfh cells isolated from LNs, higher levels were observed in splenic Tfh cells of SIV-infected RMs (at days 30 and >180) compared to naïve CD4 T cells. Furthermore, we also analyzed the expression of T-bet and Eomes, which are critical Th1 transcriptional factors. T-bet was drastically increased in Tfh cells isolated from LNs at days 30 and >180 post-infection as well as in Tfh cells isolated from the spleen although to a lower extent. In opposition, Eomes expression remained unchanged both in Tfh cells isolated from LNs and spleen (Fig 4E).

To evaluate the expression of IL-21, which is characteristic of Tfh cells, we sorted splenic Tfh cells by flow cytometry following the strategy displayed on the supplementary data (S3 Fig). *IL-21* mRNA was quantified by RT-PCR. The higher expression of the *IL21* mRNA in sorted Tfh confirmed their identity in comparison to naïve or central memory CD4 T cells (Fig 4F). Interestingly, we did not observed a difference in the levels of IL-21 expression between uninfected and SIV-infected RMs suggesting that despite higher levels of Foxo1 and KLF2, they have no impact on *IL-21* mRNA expression. In some individuals, particularly at day >180 post-infection, *IL-21* mRNA was also detected in non-Tfh cell subset. These results are consistent with a loss of Tfh cells during SIV-infection rather than the absence of IL-21 expression.

Altogether, these results demonstrated the lack of sustained expression of transcription factors required for Tfh differentiation associated with an increase in the expression of inhibitory transcriptional factors. Thus, the pool of splenic Tfh cells expressing IL-21 is decreased during AIDS.

### Loss of splenic memory B cells in SIV-infected RMs

**Fig 5:**
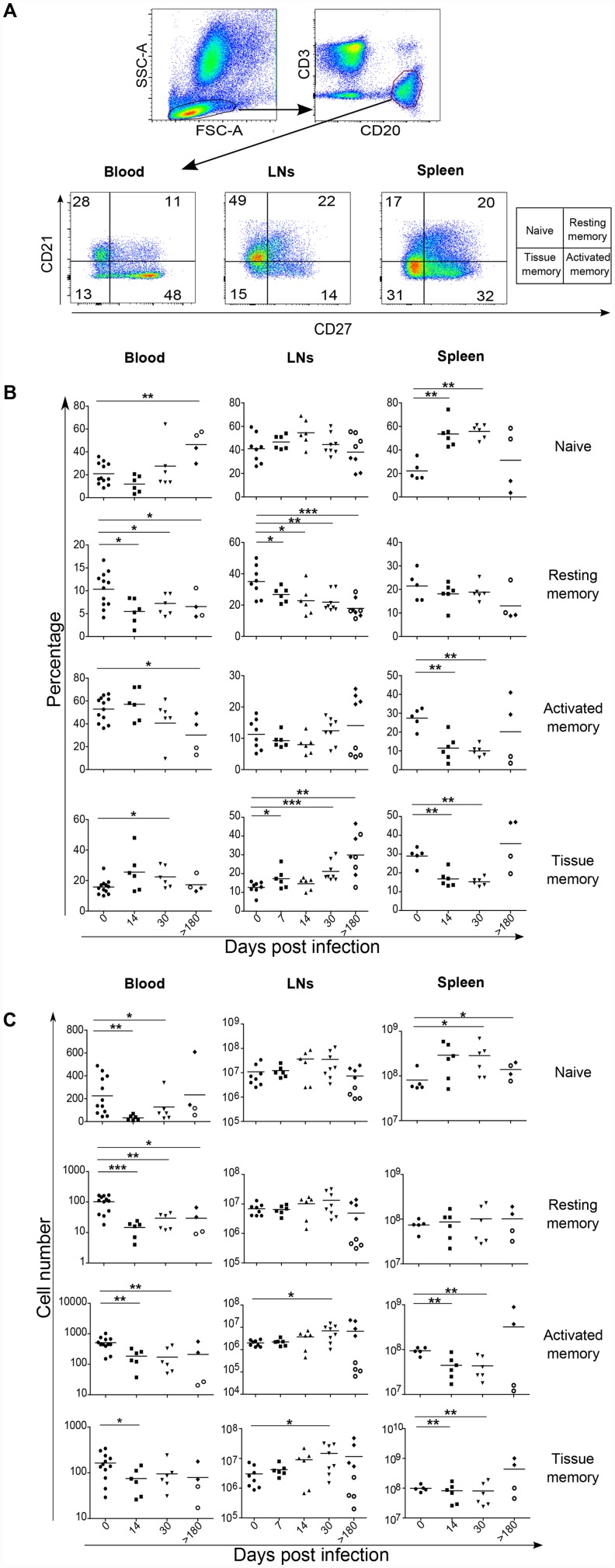
B cell dynamics in SIV-infected rhesus macaques. (A) Representative dot plots depicting gating strategy and the expression of CD21 and CD27 in B cells (CD3^−^CD20^+^) derived from blood, LNs and spleen is showed. B cell subsets were defined as naive (CD21^+^CD27^−^), resting memory (CD21^+^CD27^+^), activated memory (CD21^−^CD27^+^) and tissue memory (CD21^−^CD27^−^) B cells. (B) Percentages and (C) cell numbers for each B cell subset are shown. Each dot represents an individual RM. Statistical analyses were performed using Mann Whitney test. *, p<0.05; **, p<0.01. At day>180, open circles represent fast progressor RMs PB023 and PB028; and full diamonds represent slow progressor RMs PB013 and PB044. Axillary and Inguinal LNs are represented separately by two distinct dots for each RMs at day>180.

As Tfh cells are essential for B cell differentiation, and given the early loss of Tfh cells in the spleen compared to LNs, we analyzed the dynamics of B cell differentiation in distinct compartments during the course of SIV infection. We characterized B cell subsets by flow cytometric analysis based on the expression of CD21 and CD27 on CD20^+^CD3^-^ cells. Hence we defined naive B cells as CD21^**+**^CD27^**-**^, resting memory as CD21^**+**^CD27^**+**^, activated memory as CD21^**-**^CD27^**+**^, and tissue memory as CD21^**-**^CD27^**-**^ cells (Fig 5A). Consistent with previous reports [52–57], we found in peripheral blood lower percentages of resting and activated memory B cells (Fig 5B, left panel) in SIV-infected RMs compared to the percentages before infection.

Our results indicated that while the percentage of splenic naive B cells significantly increased early after infection (day 14, 53.6% ± 11.36% compared to day 0, 22.18% ± 8.17%; p = 0.0022), both the percentage of activated memory B cells (day 14, 11.48% ± 6.78% compared to day 0 = 27.38% ± 5.38%; p = 0.0043) and tissue memory B cells (day 14, 16.80% ± 4.22%; compared to day 0, 28.98% ± 4.68%; p = 0.0043) decreased (Fig 5B, right panel). It is noteworthy that the percentage of naive B cells remained elevated in fast progressor RMs at the chronic phase (day>180 p.i), consistent with lower levels of memory B cells, whereas the opposite balance is observed in both slow progressor RMs PB013 and PB044. Consistent with B cell lymphopenia, the pool of memory B cells diminished in the spleen of SIV-infected RMs early after infection and persisted thereafter, but again not in slow progressor RMs, PB013 and PB044 (Fig 5C, right panel).

In LNs, at day 30, a decrease in the percentage of resting memory B cells (21.89% ± 6.18% compared to day 0, 35.08% ± 9.95%; *p* = 0.0023) is observed associated with higher levels of tissue memory B cells (day 30, 21.18% ± 5.30% compared to day 0, 12.65% ± 3.18%; *p<*0.0001) (Fig 5B, middle panel). Most importantly, and consistent with the dynamics of Tfh, the percentages and the numbers of both activated and tissue memory B cells were higher in PB013 and PB044 compared to PB023 and PB28 (Fig 5C, middle panel).

**Fig 6:**
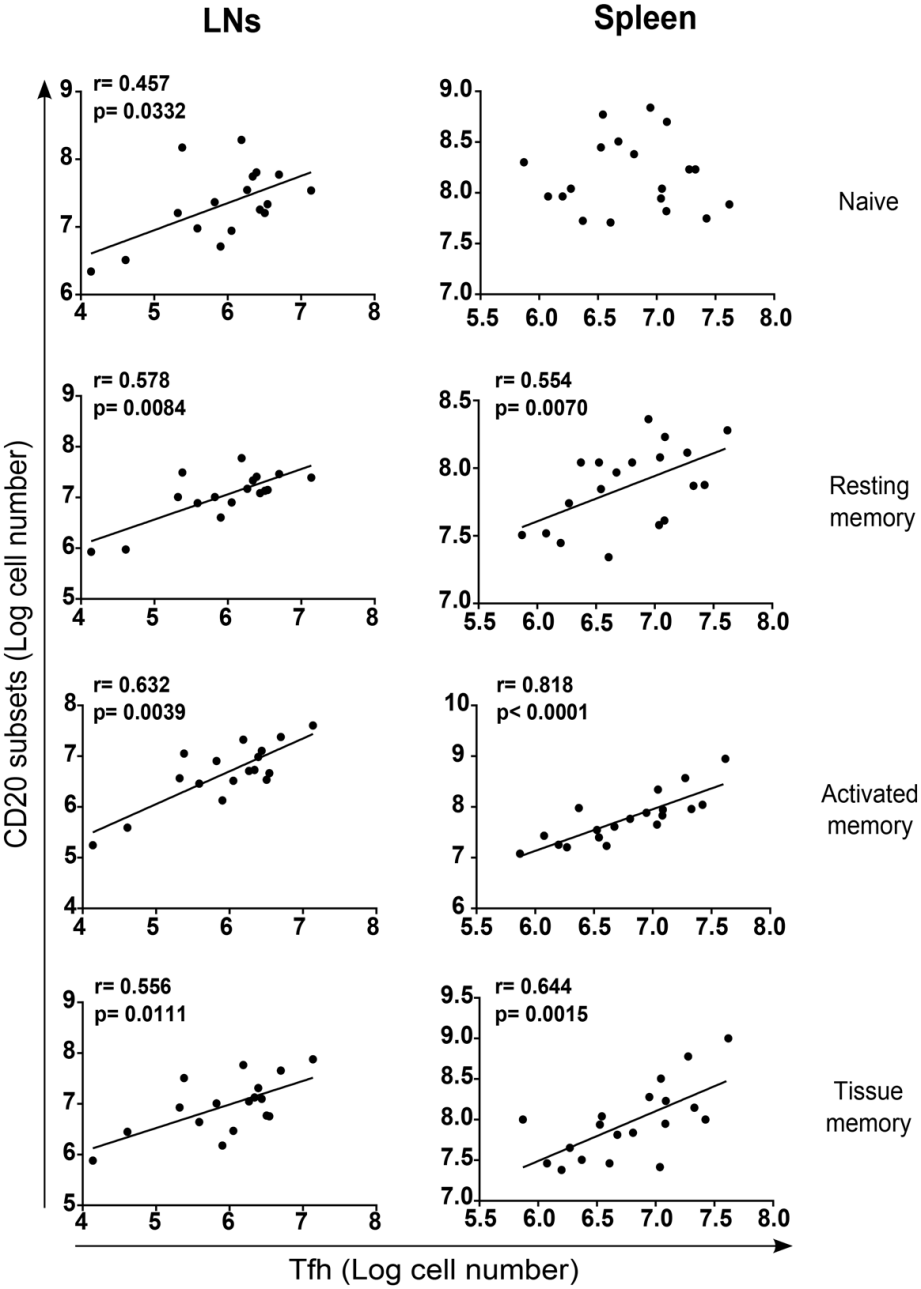
Correlation between Tfh cells and memory B cells. Tfh cell numbers (Log) were plotted against B cell subset numbers (Log) in LNs and spleen of RMs. Each dot represents an individual RM at the time of death. Spearman analysis was used for correlations. The r and p values are indicated in the figures.

**Fig 7:**
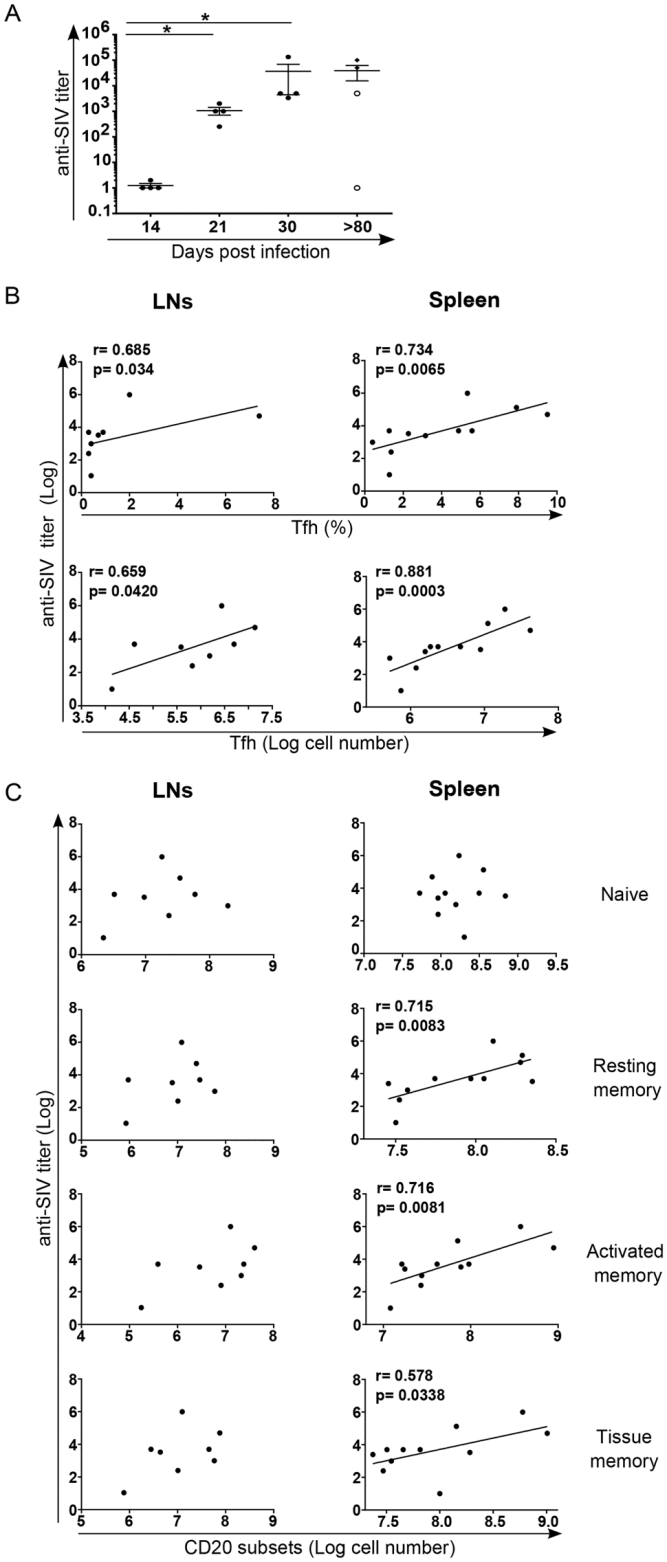
Correlation between SIV-specific IgG titers and Tfh cells. (A) Histogram shows the titer of SIV-specific IgG antibodies in the serum of SIV-infected RMs at the indicated time. At day>180, open circles represent fast progressor RMs PB023 and PB028; and full diamonds represent slow progressor RMs PB013 and PB044. (B) Dot-plots show the correlation between the titer of SIV-specific IgG antibodies and the percentage as well the number of Tfh cells in LNs and spleen of RMs. (C) Dot-plots show the correlation between the titer of SIV-specific IgG antibodies and the number of B cell subsets in LN and the spleen. Spearman analysis was used for correlations. The r and p values are indicated in the figures.

We next assessed the relationship between splenic Tfh pool and of effector memory B cell subsets. Our results clearly indicate a positive correlation between B cell differentiation and Tfh numbers in both LNs and the spleen (Fig 6), particularly with splenic activated memory B cells. In LNs, a better correlation was observed between EM T cells and B cell subsets (S4 Fig). To address the potential impact on the magnitude and quality of the developed humoral response we then assessed titers of SIV-specific IgG antibodies in SIV-infected RMs. SIV-specific antibodies were detected at day 21 post-infection and increased thereafter; no antibodies were detected at day 14 (Fig 7A). Furthermore, no SIV-specific antibodies were detected in PB023, and titer was lower in PB028 compared to PB013 and PB044 (Fig 7A). We plotted the titers of SIV-specific IgG antibodies against the percentage and the pool of Tfh cells. A strong correlation was observed with splenic Tfh cells. Although still significant, this correlate was lower with LNs cells (Fig 7B). We also assessed whether SIV-antibodies were correlated with the pool of B cell subsets. Our results indicate a positive correlation between the pool of splenic memory B cells and the titer of SIV-specific IgG antibodies, whereas no correlation was found with the pool of LNs B cells (Fig 7C), supporting the critical role of splenocytes in the production of SIV-specific IgG antibodies.

Altogether, our results demonstrated the drastic depletion of splenic activated and tissue memory B cells which might be related to the loss of fully mature Tfh cells associated with the production of SIV-specific antibodies.

### Progression to AIDS is associated with drastic remodeling of normal lymphoid architecture and loss of Tfh cells from B cell areas

**Fig 8:**
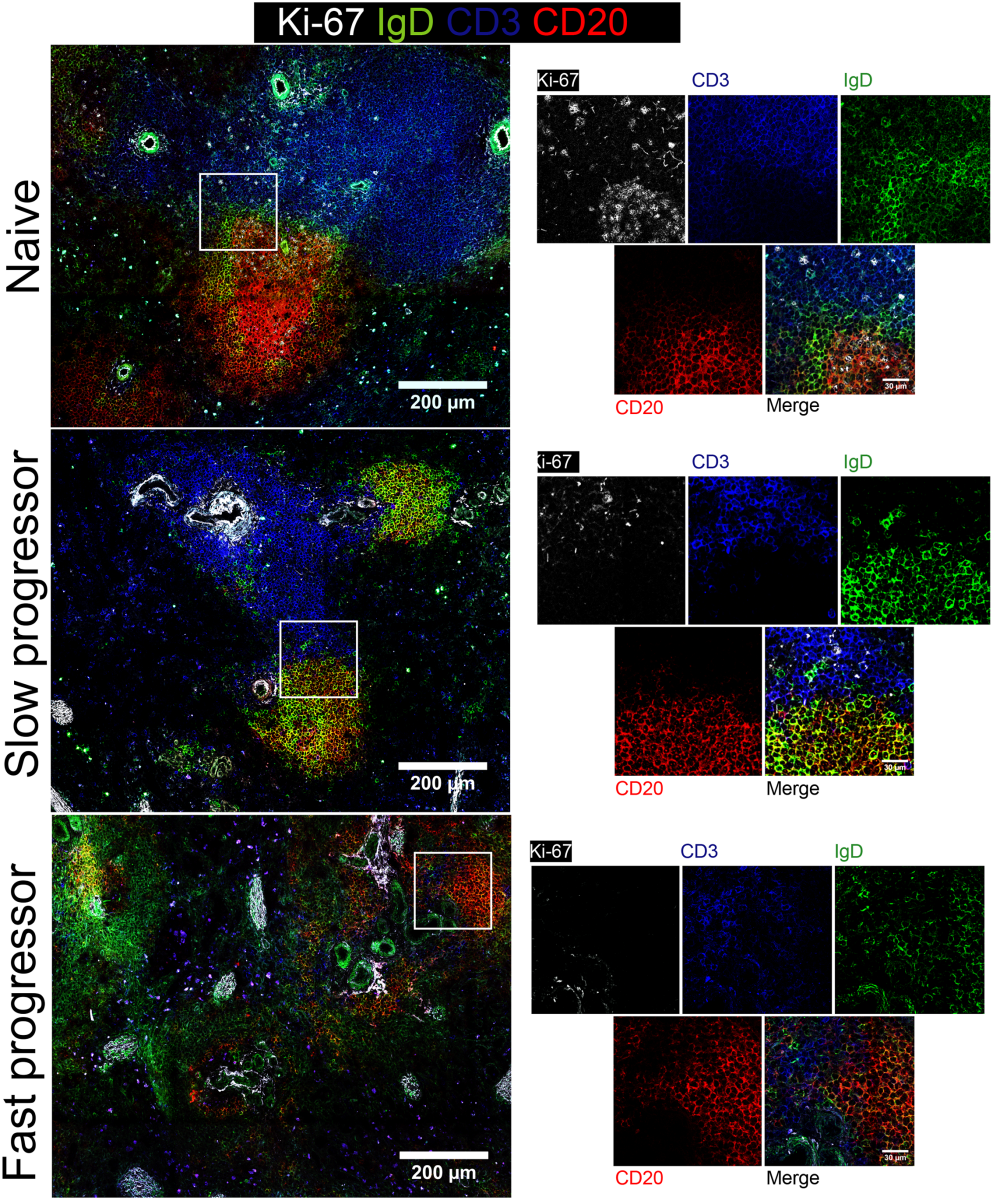
Dynamics of germinal center in the spleen of rhesus macaque infected with SIV. Splenic tissue sections were stained with antibodies against Ki-67 (white), IgD (green), CD3 (blue) and CD20 (red) and imaged by confocal microscopy. Representative pictures of a naive RM and of two chronically SIV-infected RMs, slow and fast progressor RMs are shown. The picture is representative of two individuals animals performed independently. Higher magnification is shown on the right part of the picture. Scale bar is shown.

Our data so far have highlighted the profound quantitative changes that affect the distinct lymphoid lineages during SIV infection. We next subjected splenic and LN tissue sections to confocal microscopy by analyzing T and B cell distribution aiming to elucidate whether such changes also reflected a remodeling of the lymphoid architecture. Remarkably, both the spleen (Fig 8) and LNs (S5 Fig) of a slow progressor RM demonstrated little change in the organization of their lymphoid structures as compared with non-infected counterpart (Fig 8 and S5 Fig, upper and middle panels). In stark contrast, the fast progressor RM exhibited a profound loss of normal lymphoid architecture, particularly in the spleen in which T cell area, B cell follicles and germinal centers become barely distinguishable (Fig 8, lower panels).

**Fig 9:**
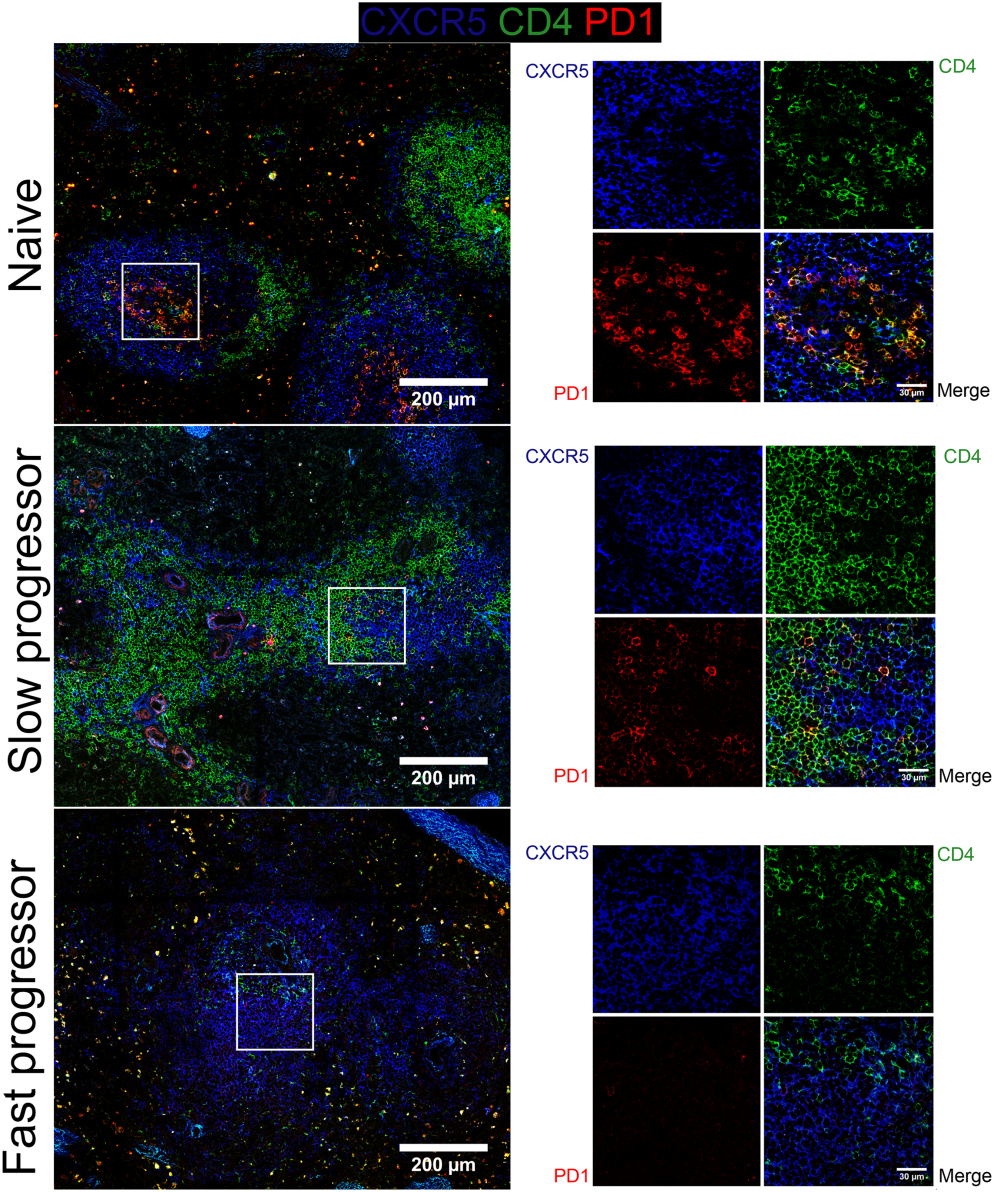
Distribution of Tfh cells in the spleen of rhesus macaque infected with SIV. Splenic tissue sections were stained with antibodies against CXCR5 (blue), CD4 (green) and PD-1 (red) and imaged by confocal microscopy. Representative pictures of the same animals as depicted in Fig 8 are shown. Higher magnification is shown on the right part of the picture. Scale bar is shown.

Given the profound impact that SIV infection has on Tfh populations, particularly in fast progressor animals, we stained splenic (Fig 9) and LNs (S6 Fig) sections to allow visualization of Tfh cells *in situ*. The pool of CD4 T cells in the spleen and LNs of non-progressor RM does not appear to be severely depleted by SIV infection as compared with naive RM (Fig 9 and S6 Fig, upper and middle panels). Furthermore, Tfh cells (CD4^+^CXCR5^+^PD-1^+^) could still be detected in B cell areas in these animals, as previously indicated by our flow cytometric analyses (Fig 2). Contrasting with slow progressor, fast progressor RM exhibits a severe depletion of CD4 T cells in both lymphoid organs and Tfh cells were hardly detectable on the B cell follicles of the spleen and LNs of these animals (Fig 9 and S6 Fig, lower panels), again in agreement with our flow cytometry data (Fig 2). Thus, confocal fluorescent microscopy confirmed the depletion of Tfh cells in lymphoid organs and highlighted the profound remodeling of the normal splenic architecture that occurs during progression to AIDS.

### Early infection of Tfh cells in SIV-infected RMs

**Fig 10:**
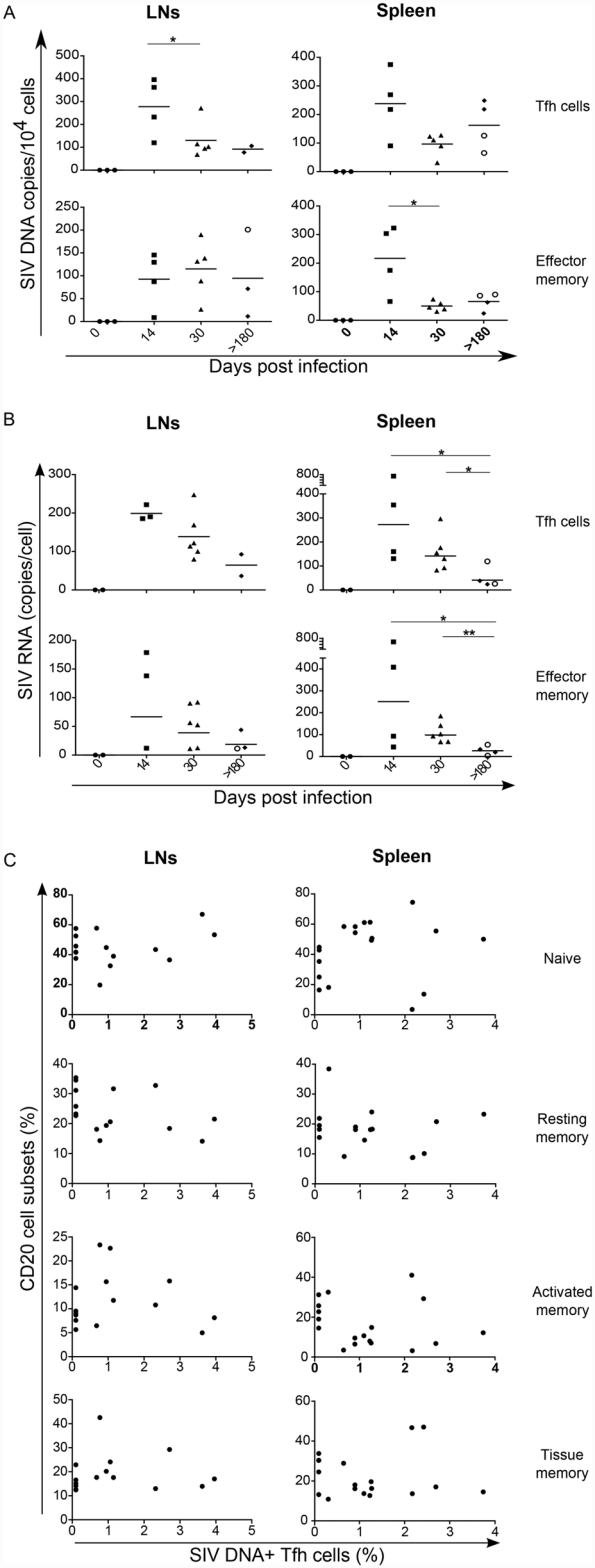
Early infection of Tfh cells. (A) Frequencies of SIV DNA and (B) RNA in sorted Tfh and effector memory CD4 T cells isolated from LNs and spleen of RMs infected with SIV. Each dot represents an individual RM. Statistical analyses are performed using Mann Whitney test. *, p<0.05; **, p<0.01. At day>180, open circles represent fast progressor RMs, PB023 and PB028; and full diamonds represent slow progressor RMs, PB013 and PB044. (C) Correlation between the frequencies of SIV DNA^+^ Tfh cells and the percentage of B cell subsets (as defined in Fig 5) in LNs and spleen of RMs. Each dot represents an individual macaque. Spearman analysis was used for correlations.

We, and others have previously reported the infection of Tfh cells both in HIV-infected individuals and in SIV-infected RMs [19,23,24]. SIV DNA and RNA were quantified from sorted Tfh and effector memory CD4 T cells. Our results demonstrated the early infection of both Tfh and effector memory CD4 T cell subsets (Fig 10A). The frequency of SIV DNA in both CD4 T cell subsets was similar in the spleen at day 14 whereas in the LNs Tfh cells displayed an increase of 3 to 4 fold comparing to effector memory CD4 T cells. At the chronic phase (>180), it is interesting to note that splenic Tfh cells of both slow progressors RMs (PB013 and PB044) harbored higher frequencies of SIV DNA than fast progressor RMs (PB023 and PB028). On the other hand, in effector memory cells SIV DNA frequencies were similar for all the animals. The pool of splenic Tfh cells in slow progressor RMs was 10 fold higher than in fast progressors (Fig 2D), suggesting that splenic Tfh cells might be a potential reservoir for SIV. Due to the low levels of Tfh cells in LNs of both fast progressor RMs SIV DNA could not be quantified. Concomitantly, we measured SIV RNA content in the sorted cells. Our data showed a peak of SIV RNA at day 14 in Tfh cells and effector memory CD4 T cells in both the spleen and LNs (Fig 10B), which decreased thereafter. Interestingly, although Tfh cells of slow progressor RMs harbored more SIV DNA than in fast progressors, the levels of SIV RNA were similar in both. In order to determine whether the infected Tfh cells may have an impact on B cells differentiation, we plotted the percentage of infected cells against the percentage of each B cell subset (Fig 10C). However, we did not observe any positive correlation between the extent of infection of Tfh cells and B cell subsets both in LNs or the spleen, suggesting that infection of Tfh cells is probably not the only main driving force causing B cell dysregulation.

Therefore, our results demonstrated the early infection of splenic Tfh cells in SIV-infected RMs, and suggested that Tfh cells could be a potential reservoir for SIV.

## Discussion

Our results revealed that splenic Tfh cells, which are expressing IL-21 mRNA, are specifically depleted early after SIV-infection in RMs. Moreover, we found an increase of the inhibitory transcriptional factors KLF2 and Foxo1 as well as an absence of sustained expression of Bcl-6 and c-Maf, which are essential for Tfh differentiation. This abortive differentiation of Tfh cells concomitantly with the alteration of the architecture of the spleen impaired their interaction with B cells leading to the loss of memory B cells, and the production of SIV antibodies. Thus, our data suggests that depletion of splenic Tfh cells in individuals may represent a critical component in HIV immunodeficiency given that the spleen represents the main organ for the initiation of B cell responses.

Whereas several groups have reported an increase of Tfh cells among memory CD4 T cells [20,23,58], other groups have observed a decreased in Tfh cells. In that sense, Boswell *et al*. [25] showed a loss of Tfh cells during HIV infection by expressing her data proportionately to the total number of CD4 T cells. Moreover, Petrovas *et al*. [21] reported that half of the chronically SIV-infected RMs had elevated LNs Tfh cells, which are accompanied by preserved architecture and distribution of Tfh and B cells in LNs. This profile associated with lower accumulation of naive CD4 T cells is a hallmark of non-progression to AIDS. Moreover, during the revision of our manuscript, two additional manuscripts reported that the quantity of Tfh is lower in LNs of progressor SIV-infected RMs compared to non-progressor SIV-infected RMs [59,60]. Thus, these results are consistent with our data showing that slow progressor RMs display an increased frequency of Tfh cells in LNs whereas their numbers drastically decreased in fast progressors RMs.

Tfh cells are characterized by their expression of CXCR5 and PD-1. Our results indicated the presence of different subsets of memory CD4 T cells expressing CXCR5 in SIV-infected RMs. In the context of the lymphocytic choriomeningitis virus (LCMV) infection distinct subsets of CXCR5 T cells have been described [41]. Whereas mostly Tfh cells are effector memory cells (CD45RA^-^CD62L^-^) before infection, Tfh cells exhibited a central effector memory phenotype (CD45RA^-^CD62L^+^) after LCMV infection. Our data demonstrated that SIV-infected RMs present a similar switch. Moreover, we observed that CXCR5 positive CD4 T cells transiently expressed higher levels Bcl-6 and c-Maf, suggesting the lack of sustained expression of these transcriptional factors [14,61]. In the context of bacterial infection, it has been reported that Tfh cells do not enter the memory pool [62]. We recently demonstrated the transient expansion of immature Tfh cells in the context of *L*. *infantum* infection, which was associated with an inability to maintain the pool of effector memory B cells and parasite-specific antibodies during chronic visceral leishmaniasis [28]. These results suggest that HIV and SIV infections are associated with abortive Tfh differentiation, which is unable to replenish the pool of Tfh cells lost early after infection. Our data also indicated for the first time in the context of viral infection that Tfh cells express KLF2 and Foxo1, which are inhibitory transcriptional factors responsible for arresting CXCR5 and Bcl-6 expression [46,47]. Thus, we interpret the observation that Tfh cells express higher levels of CD62L after SIV-infection by the fact that those transcriptional factors regulate the expression of CD62L [46,50,51]. Moreover, mRNA expression of both *CXCR5* and *MAF* transcripts were higher in slow progressors than in fast progressors of chronically SIV-infected RMs of Chinese origin [63]. These results in both Indian and Chinese macaques suggested that progression to AIDS is associated with lower levels of Tfh cells.

*In situ* analysis of CXCR5 and PD-1 expression was consistent with flow cytometry data by demonstrating the absence of fully mature splenic GC-Tfh cells (CXCR5^+^PD-1^+^) in fast progressor animals. The immature cells observed were mostly negative for PD-1. Hong *et al*. investigating the dynamics of GC formation showed that fast disease progression upon infection is associated with an involution of GCs without local IL-21 production [32]. Moreover, it was previously shown the progressive loss of IL-21 producing cells in HIV infected individuals, which remained at normal levels in slow progressors and ELITE controllers [26]. Although the percentage of Tfh cells, which express CXCR5 concomitantly with high levels of PD-1 (PD-1^bright^), is lower in peripheral blood compared to the spleen (around 20 fold less), altogether these results point to the absence of GC-Tfh cells in pathogenic HIV/SIV infections. Cubas *et al*. [20] have proposed that LNs Tfh cells of infected subjects are not intrinsically dysfunctional; yet, when in contact with PD-L1-expressing B cells their capacity to produce IL-21 is diminished. These differences may reflect distinct immune response of Tfh cells depending on tissue localization and the nature of help signals, consisting of both cytokines and cell surface molecules.

It has been also shown that some T cells, located at the interface between the T cell zone and the follicle, outside of the GCs, also express CXCR5 and produce IL-21 [64]. These cells, named pre-GC Tfh cells, appear early after immunization and are progressively replaced by PD1^bright^ CD4 T cells that locate within GCs [64–66]. Complete differentiation of Tfh cells depends on cognate interactions between primed CD4 T cells and antigen-activated B cells [67,68]. In this sense, B cells play a crucial role for the survival of Tfh cells and the commitment to the Tfh lineage [67]. In the absence of B cells, Tfh cells are still developed, even though in significantly lower numbers that fail to express PD-1 [68]. Therefore, the CXCR5^+^PD-1^−^ CD4^+^ T cell population that we detected may represent a pre-GC-Tfh state that does not mature to a bona fide Tfh population due to the lack of cognate interactions with B cells. Physical contact between Tfh and GC B cells presenting the highest levels of cognate peptide bound to major histocompatibility complex II is essential [13,69]. Interestingly, our imaging data revealed a nearly total absence of CXCR5 and/or PD-1 expressing CD4 T cells in the B cell follicles of the LNs and spleen of fast progressor RMs; yet, slow progressor RMs preserved a pool of GC-associated Tfh cells. The disruption of tissue architecture during SIV-infection is associated with increased expression of immunosuppressive cytokines such as TGF-β and IL-10 associated with collagen deposit and fibrosis [40,70–72]. In particular, we have previously reported a positive correlation between the level of TGF-β and the progression to AIDS [40,70]. Thus, this profound remodeling in animals progressing fast may impact on the capacity of those cells to be adequately activated compared to animals progressing slowly to AIDS. Therefore these observations sustained the hypothesis that during SIV-infection abnormal bi-directional communication in lymphoid organs between B and T cells may participate in a defective immune response against microbes.

Due to the critical interaction between Tfh and B cells, the depletion of Tfh cells in the spleen very early after infection may participate in the absence of maturation and loss of memory B cells. It is well known that HIV infection leads to chronic B cell immune activation in individuals with AIDS [73]. There are also some indications that impairment of B-cell function occurs early after infection [52,74,75]. It has been shown that B cells are abnormally prone to undergo apoptosis through engagement of PD-1 [53–55] and death-receptors [56,57]. Interestingly, it has been also shown that the depletion of B cells leads to the death of SIV-infected Pigtail macaques implying that humoral response is an important protection factor [76]. Whereas the vast majority of the results concern peripheral blood and LNs, paradoxically visceral tissues are poorly documented. Herein we demonstrated the progressive loss of memory B cells in the spleen and the accumulation of naive B cells, which is consistent with a previous report in SIV-infected cynomolgus macaques [77]. Moreover, consistent with the reciprocal role of Tfh and memory B cells, we found a positive correlation between the numbers of Tfh and memory B cell subsets in the spleen. Thus, in the context of defect of co-signals provided by close contact with Tfh cells, memory B cells can be abnormally primed to die [78,79]. In this context, it is interesting to note that we and others have reported that the level of B-cell activation within the GC is higher in non-pathogenic SIV-infected African green monkey (AGMs) than in pathogenic SIV-infected Chinese or Indian RMs [63,80,81]. Indeed, our data demonstrates that SIV-specific IgG titers were higher in slow progressors than in fast progressor RMs. Moreover, our data clearly indicate a strong correlation between SIV-specific IgG and the pool of splenic Tfh cells. In HIV-infected individuals a reduction in antibody responses against hepatitis B, measles, mumps and rubella vaccines were reported [52,82]. Furthermore, patients who are responders to a Flu vaccine display an expansion of Tfh cells compared to non-responders [83], supporting a role of Tfh cells in maintaining the pool of long live memory B cells. Finally, it is conceivable to envision that, by inducing an early depletion of Tfh in the spleen, SIV-infection promotes inappropriate B cell differentiation and favors B cell death. Such abnormal B cell differentiation will, in turn, impact on Tfh development resulting in compromised numbers of Tfh population to sustain GC responses in the chronic phase of the infection. The results included in the current study strongly suggest that the depletion of Tfh cells in the spleen is an early pathogenic event associated with AIDS.

Growing evidence suggests that Tfh cells are infected by HIV/SIV early after infection [21,24]. Here, we demonstrated the early infection of Tfh cells both in peripheral LNs and the spleen. However, it is noteworthy that the frequencies of SIV DNA^+^ Tfh cells as well as their numbers were higher at the chronic phase in slow progressors than in fast progressors RMs suggesting that immune control restrain or maintain the virus within the B cell follicle regions. Therefore, this subset represents a possible viral reservoir in immune controllers. We and others have previously reported that SIV is present in the GCs [63,84]. Importantly, trapping of SIV in GCs was also observed in non-pathogenic SIV-infected AGMs [63] or in sooty mangabey at the border of the GCs where the Tfh cells are localized [85]. Nevertheless, the amount of viral particles trapped in the region of GCs increases with the pathogenicity [84,86]. Despite their high frequency in SIV DNA, Tfh cells of slow progressors showed a similar or lower level of cellular SIV RNA compared to fast progressors, pointing to the fact that slow progressor Tfh cells might be less active to replicate SIV than Tfh cells of fast progressor. However, we did not find any correlation between the extent of SIV-DNA^+^ Tfh cells and the percentages of memory B cell subsets, suggesting that infection of Tfh population by itself is not directly associated with abnormal B cell differentiation. Because viruses are trapped in the GCs where follicular dendritic cells are present, this can affect B cell differentiation [87]. Similarly, soluble HIV proteins such as nef have been reported to interact with B cells either directly [88] or indirectly [89,90]. Therefore, these factors in addition to Tfh cell loss could participate in the dysregulation of B cell response.

Given that the Tfh population is a subset of memory CD4 T cells, which have been shown to be highly prone to die by apoptosis and that the extent of apoptosis is predictive of further progression towards AIDS characterizing pathogenic lentiviral infections, depletion of splenic Tfh cells may be related to the occurrence of apoptosis [33,34,36,38,40,43,44,91–94]. In this sense, Petrovas *et al*. [21] have shown higher propensity of Tfh cells to undergo apoptosis based on the expression of the caspase-3, an effector of apoptosis, and have limited proliferative capability [19]. Tfh cells express CD95 and because CD95L have been shown to be elevated in pathogenic compared to non-pathogenic infections [95], and are involved in the death of memory CD4 T cells [36–38,96], it cannot be excluded that FasL is directly involved in the death of Tfh cells. Furthermore, our data indicated the accumulation of Tfh cells displaying a central memory phenotype instead to an effector memory phenotype; this former being less prone to die than the latter. This scenario is compatible with preservation of the pool of Tfh cells in RMs, despite their high infection rate.

In conclusion we have demonstrated the early loss and infection of splenic Tfh cells, which is associated with the loss of memory B cells. The lack of sustained expression of transcription factors governing Tfh differentiation certainly participates in the absence of full maturation and reconstitution of the pool of Tfh cells. The huge remodeling of the splenic architecture also restrained the capacity of Tfh and B cells to interact, which is essential for both. Altogether, these may contribute to the poor immune control occurring in fast progressor RMs. Therefore, strategies aimed to prevent the loss of Tfh cells or to help the reconstitution of splenic Tfh cells could be useful for individuals but also for vaccine strategy. However, because Tfh cells are infected early, this subset may represent a reservoir, which may impair functional HIV cure.

## Materials and Methods

### Ethics statement

All animals were housed at University Laval in accordance with the rules and regulations of the Canadian Council on Animal Care (http://www.ccac.ca). This protocol was approved by the Laval University Animal Protection Committee (Project number 106004). Animals were fed standard monkey chow diet supplemented daily with fruit and vegetables and water ad libitum. Social enrichment was delivered and overseen by veterinary staff and overall animal health was monitored daily. Animals showing significant signs of distress, disease, and weight loss were evaluated clinically and were humanely euthanized using an overdose of barbiturates according to the guidelines of the Veterinary Medical Association.

### Animal, viral inoculation and sample collection

Rhesus macaques (*Macaca mulatta*) of Indian origin, seronegative for SIVmac, STLV-1 (Simian T Leukemia Virus type-1), SRV-1 (type D retrovirus) and herpes-B viruses were used in this study. Sixteen animals were infected with SIVmac251 virus (20 AID_50_) intravenously and five were left as non-infected controls. Subgroups of animals were euthanized at different time points post-infection covering both acute and chronic phases. Peripheral blood and lymphoid organs (spleen and axillary and inguinal lymph nodes) were recovered for cellular analysis. Cell numbers were calculated from LNs retrieved in each region (inguinal and axillary LNs, the totality of the LNs were retrieved). Cells isolated after mechanical process were counted. Tissues were not digested with collagenase or other proteases for cell isolation limiting side effects on the expression of cell surface markers. Blood sampling was performed at additional time points before and after infection. For each blood-sampling point, a hemogram was elaborated using an Abaxis VetScan HM5 hematology instrument (Abaxis, CA). Frozen samples derived from the spleen of SIV-infected RMs of Chinese origin of previous studies [36,40,70] were used to assess mRNA expressions.

### Quantitative-PCR

Samples from rhesus macaques (*Macaca mulatta*) of Chinese origin infected either with *Leishmania infantun* promastigotes or SIVmac251 [28,45] were also used to assess mRNA expression of *CXCR5*, *BCL6*, and *MAF*. Approximately 10 millions of splenic mononuclear cells or total peripheral LNs cells were lysed in RLT buffer (RNeasy Micro Kit, QIAGEN) and stored at -80°C until further use. RNA was purified using the RNeasy Micro Kit following manufacturer’s instructions and reverse transcribed using the Affinity Script QPCR cDNA synthesis kit (Stratagene). Gene expression was analyzed by qPCR in 10 μL reactions, using 100 ng of cDNA. The thermal profile consisted of a hold of 15 min at 95°C, followed by 40 cycles of denaturation (95°C, 15 sec), annealing (60°C, 30 sec) and extension (72°C, 30 sec). Ct values were normalized by quantifying the levels of GAPDH or 18S. Macaque-specific primers were designed using the AutoPrime software. A list of primer sequences used is provided in S1 Table.

### Cell sorting

10^8^ freshly isolated cells were sorted using BD Influx cell-sorter based on their expression of CD3, CD8, CD4, PD-1, CXCR5, CD45RA and CCR7. Cells were separated in five different subsets, naïve, central memory, effector memory, differentiated effector memory and T follicular helper cells as shown in S3 Fig. Samples were preserved at -80°C pelleted until used for DNA analysis and in Trizol (Life Technologies) for RNA purification.

### Proviral DNA quantification

DNA was purified from 10^5^sorted Tfh and effector memory cells from spleen and LNs using the Genomic DNA Tissue kit from (Macherey Nagel). The cell line SIV-1C, that contains one copy of SIVmac251 DNA per cell was used as reference for proviral DNA quantitation. Serial dilutions of SIV-1C cells with CEMX174 carrier cells were performed to generate a standard curve. SIV-proviral DNA was amplified by nested PCR with SIVmac251-specific primers surrounding the nef region. A first round of PCR was performed using 50 nM of preco and K3 primers, 10X PCR buffer, 0.8 mM DNTP, 2 mM MgCl_2_, 1.25 U of AmpliTaq Gold (Life Technologies) in a Biometra thermocycler using the following parameters: one cycle of 105 sec at 95°C, 45 cycles of 30 sec at 95°C, 30 sec at 60°C and 10 sec at 72°C, 6 min at 72°C. The PCR product was diluted 1/8 and 5 μl were re-amplified using 250 nM of A2 and K1 primers and 2X Quantitec Sybr Green PCR Kit (Qiagen) in a 7500 Real-Time PCR System (Applied Biosystems), using the following parameters: one hold of 2 min at 50°C, 15 min at 95°C and 45 cycles of 15 sec at 94°C, 30 sec at 60°C and 35 sec at 72°C. Ribosomal 18S DNA was amplified in parallel as an internal control. A standard curve was used to estimate cell numbers, and the results were expressed as SIV proviral DNA copies per 10,000 cells.

### Viral RNA quantification

RNA was purified from 10^5^ sorted cells kept in Trizol (Life Technologies) according to the manufacturer’s instructions. Viral RNA was extracted from 200 μl of plasma with PureLink Viral RNA/DNA Kit (Life Technologies). RNA was treated with Turbo-DNA free kit (Life Technologies) and quantified by RT-qPCR using 4X TaqMan Fast Virus 1-Step Master Mix (Life Technologies), 750 nM of primers and 200 nM of probe (S1 Table). 10-fold serial dilutions of a SIVmac251 plasmid including SIV *gag* gene were performed to generate a standard curve, starting at 10^9^ SIV genome copies/μl. Amplifications were carried out with a *7500* Real-Time PCR System (Applied Biosystems), using the following parameters: 50°C /5 min, 95°C /20 sec, 40 cycles (95°C /15 sec, 60°C /1 min). 18S rRNA was used as endogenous control using the Eukaryotic 18S rRNA Endogenous Control mix (Life Technologies) for cell samples. Results are expressed as SIV RNA copies per cell detected in cell samples or copies per μl in serum samples.

### Immunophenotyping

Fresh cell suspensions were prepared from macaque spleen, axillary and inguinal LNs. Peripheral blood was collected to EDTA-coated tubes. Fresh cells were stained with a panel of monoclonal antibodies. The fluorochrome-conjugated antibodies used are provided in the S2 Table. Intracellular Bcl-6, c-Maf, Foxo1, KLF2, Eomes, and T-bet staining was performed after fixing and permeabilizing the cells with the FoxP3 staining buffer set (eBioscience). After lysing erythrocytes (Lysing buffer Pharm Lyse 10X BD Biosciences), sixty thousand events corresponding to mononuclear cells were recorded in FACS Canto A (BD Bioscience). Analyses were performed using FlowJo software (Tree Star, Inc.).

### Immunofluorescence confocal microscopy of tissue sections

Tissues (spleen and peripheral lymph nodes) were embedded in optimal cutting temperature compound (OCT), sectioned at a 7.5 μm thickness and stored unfixed at -80°C until use. Tissue sections were fixed in 4% PFA (15 minutes at room temperature) followed by acetone (20 minutes at -20°C). Slides were submerged in blocking solution (5% normal goat serum, 0.3% triton X-100) for 1 hour at RT.

For this work, we took advantage of two multicolor panels that we had optimized previously [28]. For Panel 1 (Ki-67/IgD/CD3/CD20), sections were initially incubated overnight at 4°C with a purified anti-Ki-67 antibody diluted in antibody dilution buffer (1% BSA, 0.3% triton X-100). The next day, sections were washed and incubated with a secondary antibody-coupled to Brilliant Violet-421 (BV-421) diluted in antibody dilution buffer for 1 hour at room temperature. After thorough washing, sections were incubated overnight at 4°C with fluorochrome-coupled antibodies against the other markers of the panel; IgD-FITC, CD3-PECF594 and CD20-AF647. After washing, slides were mounted with antifade mounting medium.

The procedure employed for Panel 2 staining (CXCR5/CD4/PD1) was similar. Briefly, sections were initially incubated with a purified anti-CXCR5 antibody overnight, followed by a secondary antibody coupled to BV421 (The following reagent was obtained through the NIH Nonhuman Primate Reagent Resource: CXCR5). After washing, samples were incubated overnight with directly coupled antibodies for the remaining markers; CD4-PECF594 and PD1-AF647. S2 Table provides detailed information on the antibodies used for tissue immunofluorescence. Sections were imaged in a Zeiss LSM 710 confocal microscope. Tiled Z-stacks were acquired with a 20X objective and stitched using the Image J stitching plugin [97]. Average intensity projections were obtained from the stitched tiles using built-in Image J tools.

### Detection of anti-SIV specific IgG

Specific IgGs were detected in serum samples using a homemade HIV-2 ELISA. Briefly, inactivated HIV2 crude extract (Biorad) was coated at 0.5 μg/per well. Serial dilutions of serum samples were incubated in HIV-2 antigen-coated plates to determine the serum titer. IgGs were detected using a goat anti-monkey IgG HRP antibody (Serotec). Results are expressed as titer of SIV-specific IgG antibody.

### Statistical analysis

Statistics were performed with the GraphPad Prism 5 software. The non-parametric Mann Whitney test was employed for comparison between naive and infected animals at different time points after infection. A spearman test was employed for correlations.

## Supporting Information

S1 Fig CD3 T cell dynamics in SIV-infected rhesus macaques. (A) Representative dot plots depicting the expression of CD3 and CD20 in the blood. (B) Histograms show percentages and cell numbers of CD3 T cells in the blood (number/mm^3^), LNs and spleen of RMs at the indicated time. Statistical analyses are performed using Mann Whitney test. *, p<0.05; **, p<0.01. At day>180, open circles represent fast progressor RMs PB023 and PB028; and full diamonds represent slow progressor RMs PB013 and PB044.
(TIF)

S2 Fig Dynamics of CD4 T cell subsets in SIV-infected rhesus macaques. (A) Representative dot plots depicting the expression of CD62L and CD45RA in the blood, LNs and the spleen of non-Tfh cells. (B) Histograms show the expression of PD-1 for each subpopulation: naive (CD45RA^+^CD62L^+^), central memory (CM, CD45RA^-^CD62L^+^), effector memory (EM, CD45RA^-^CD62^-^), and terminally differentiated (TDT, CD45RA^+^CD62L^-^). (C) Percentage and (D) cell number of naive, central memory and terminally differentiated CD4 T cells in the blood, in LNs and spleen. Each dot represents an individual RM. Statistical analyses are performed using Mann Whitney test. *, p<0.05; **, p<0.01. At day>180, open circles represent fast progressor RMs PB023 and PB028; and full diamonds represent slow progressor RMs PB013 and PB044.
(TIF)

S3 Fig Cell sorting strategy. Representative dot plots depicting gating strategy used to sort CD4 T cell subsets using BD influx cell sorter. CD3^+^ T cells are separated in CD3^+^CD4^+^ and CD3^+^CD8^+^ T cells. After gating on CD3^+^CD4^+^, Tfh cells are sorted based on the expression of CXCR5 and PD-1 (CXCR5^+^PD-1^bright^). Non-Tfh CD3^+^CD4^+^ T cells are then separated in naive (CD45RA^+^CCR7^+^), central memory (CD45RA^−^CCR7^+^), effector memory (CD45RA^−^CCR7^−^) and terminally differentiated (CD45RA^+^CCR7^−^).
(TIF)

S4 Fig Correlation between effector memory CD4 T cells and B cell subsets. Diagrams show correlation between the percentage of effector memory cells and the percentage of B cell subset (as defined in fig 5) in LNs and spleen of RMs. Each dot represents an individual RM. Spearman analysis was used for correlations.
(TIF)

S5 Fig Dynamics of germinal center in LNs of rhesus macaque infected with SIV. LN tissue sections were stained with antibodies against Ki-67 (white), IgD (green), CD3 (blue) and CD20 (red) and imaged by confocal microscopy. Representative pictures of a naive RM and of two chronically SIV-infected RMs, slow and fast progressor RMs are shown. The picture is representative of two individuals animals performed independently. Higher magnification is shown on the right part of the picture. Scale is shown.
(TIF)

S6 Fig Distribution of Tfh cells in LNs of rhesus macaque infected with SIV. LNs tissue sections were stained with antibodies against CXCR5 (blue), CD4 (green) and PD-1 (red) and imaged by confocal microscopy. Representative pictures of the same animals as depicted in S5 Fig are shown. Higher magnification is shown on the right part of the picture. Scale is shown.
(TIF)

S1 Table Primers and probes used for RT-qPCR. (PNG)

S2 Table Antibodies used for flow cytometry, cell sorting and immunofluorescence. Flow cytometry and cell sorting (upper list), tissue immunofluorescence (bottom list).
(PNG)
