# Correction: Early Loss of Splenic Tfh Cells in SIV-Infected Rhesus Macaques

**DOI:** 10.1371/journal.ppat.1005393

**Published:** 2016-01-13

**Authors:** Félicien Moukambi, Henintsoa Rabezanahary, Vasco Rodrigues, Gina Racine, Lynda Robitaille, Bernard Krust, Guadalupe Andreani, Calayselvy Soundaramourty, Ricardo Silvestre, Mireille Laforge, Jérôme Estaquier

There are errors in the Funding section. The correct funding information is as follows: This work was supported by grants from ANRS (France), the Canadian Institutes of Health Research (CIHR) (HBF-123682, HBF-126786, and MOP-133476), and by the Canadian HIV Cure Enterprise Grant HIG-133050 from the CIHR partnership with CANFAR and IAS to JE. JE thanks the Canada Research Chair program for financial assistance. VR was supported by a doctoral fellowship from FCT (Fundação para a Ciência e Tecnologia); code SFRH/BD/64064/2009. We would like to thank the Nonhuman Primate Reagent Resource for kindly providing CXCR5 antibodies. The funders had no role in study design, data collection and analysis, decision to publish, or preparation of the manuscript.
