# Supplementary figures and images for: Early Loss of Splenic Tfh Cells in SIV-Infected Rhesus Macaques
Source: PLoS Pathog. 2015 Dec 7;11(12):e1005287. doi: 10.1371/journal.ppat.1005287 (PMC4671657; doi:10.1371/journal.ppat.1005287)

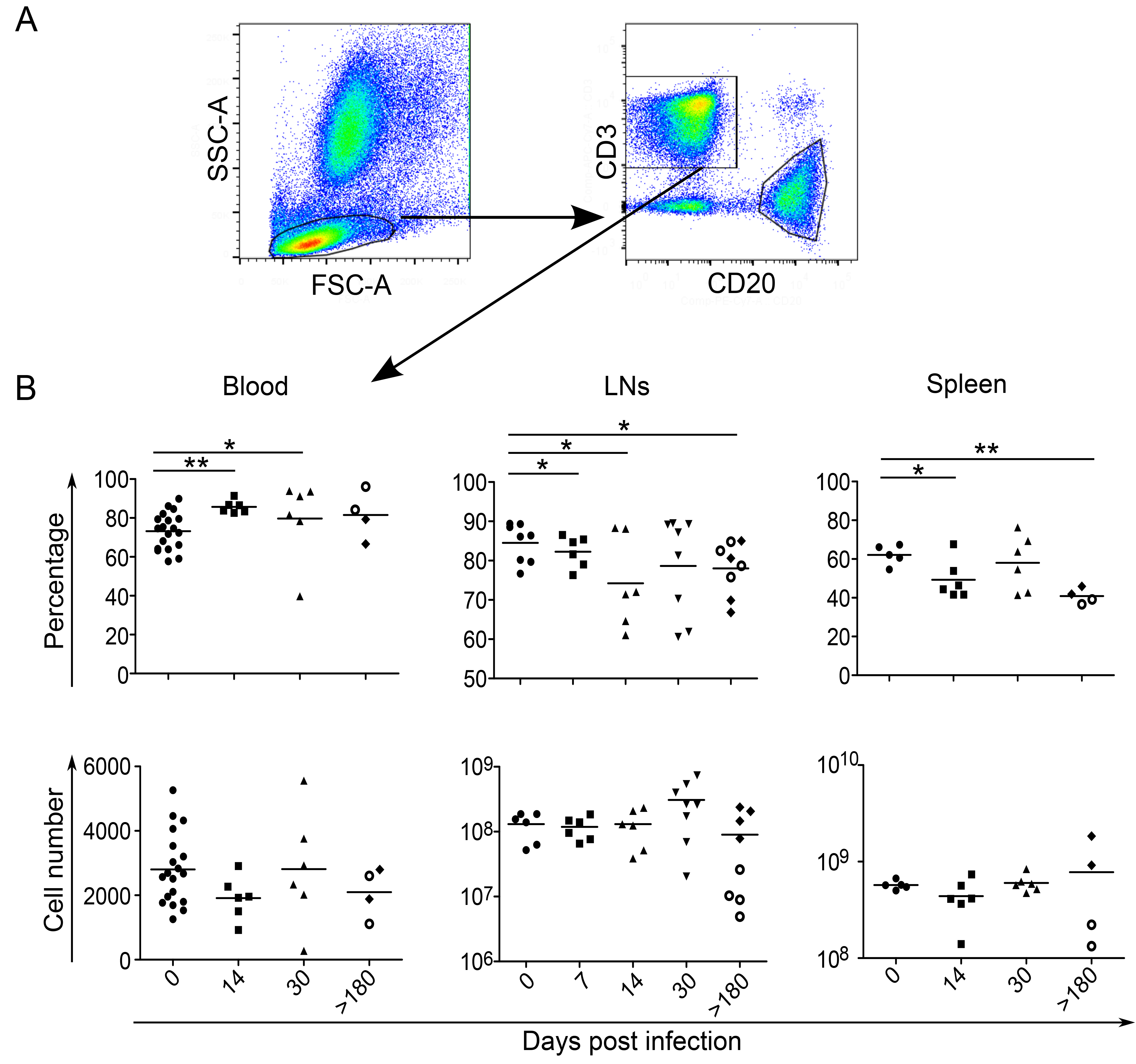

Supplement: S1 Fig — (A) Representative dot plots depicting the expression of CD3 and CD20 in the blood. (B) Histograms show percentages and cell numbers of CD3 T cells in the blood (number/mm3), LNs and spleen of RMs at the indicated time. Statistical analyses are performed using Mann Whitney test. *, p<0.05; **, p<0.01. At day>180, open circles represent fast progressor RMs PB023 and PB028; and full diamonds represent slow progressor RMs PB013 and PB044. (TIF) [file ppat.1005287.s001.tif]

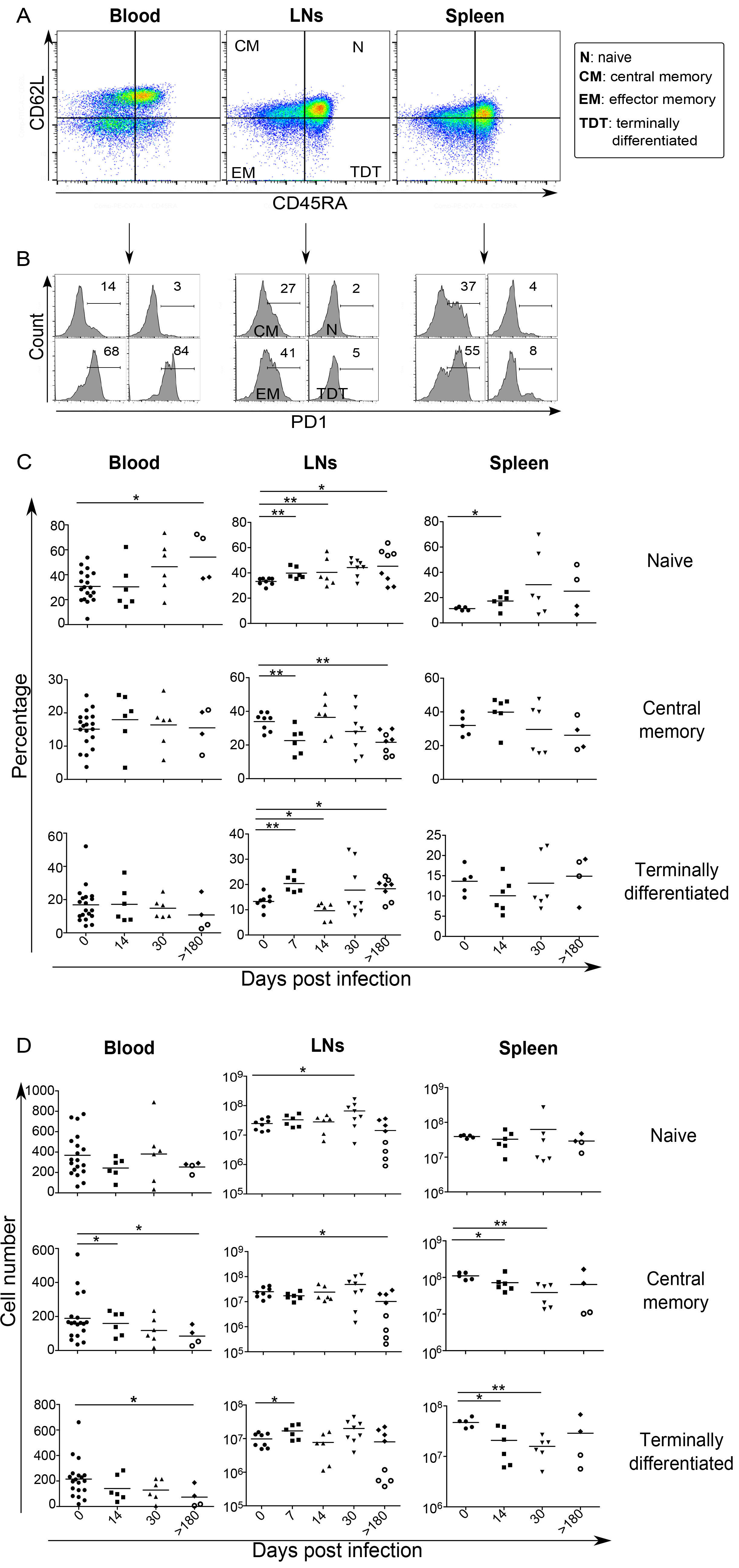

Supplement: S2 Fig — (A) Representative dot plots depicting the expression of CD62L and CD45RA in the blood, LNs and the spleen of non-Tfh cells. (B) Histograms show the expression of PD-1 for each subpopulation: naive (CD45RA+CD62L+), central memory (CM, CD45RA-CD62L+), effector memory (EM, CD45RA-CD62-), and terminally differentiated (TDT, CD45RA+CD62L-). (C) Percentage and (D) cell number of naive, central memory and terminally differentiated CD4 T cells in the blood, in LNs and spleen. Each dot represents an individual RM. Statistical analyses are performed using Mann Whitney test. *, p<0.05; **, p<0.01. At day>180, open circles represent fast progressor RMs PB023 and PB028; and full diamonds represent slow progressor RMs PB013 and PB044. (TIF) [file ppat.1005287.s002.tif]

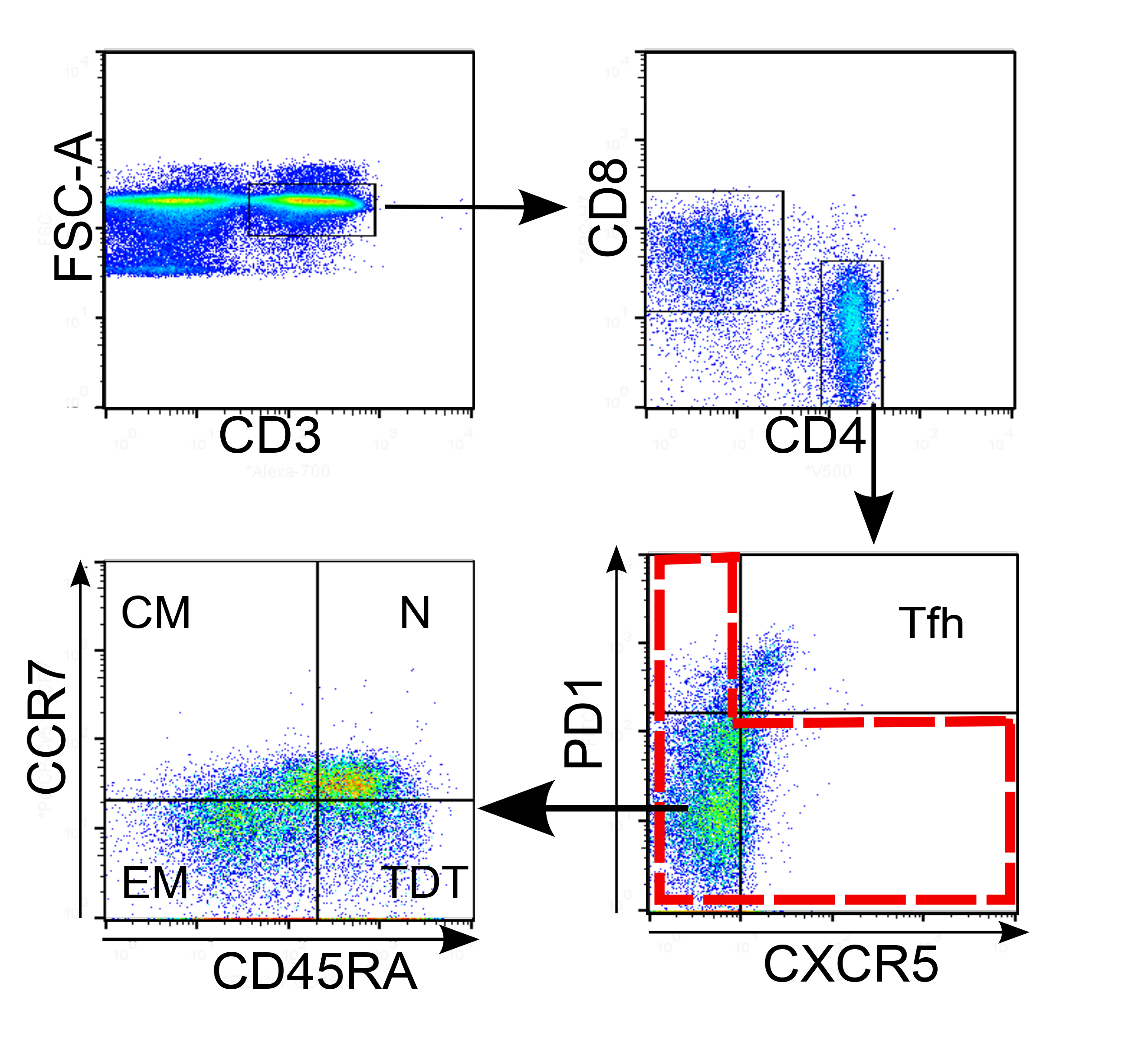

Supplement: S3 Fig — Representative dot plots depicting gating strategy used to sort CD4 T cell subsets using BD influx cell sorter. CD3+ T cells are separated in CD3+CD4+ and CD3+CD8+ T cells. After gating on CD3+CD4+, Tfh cells are sorted based on the expression of CXCR5 and PD-1 (CXCR5+PD-1bright). Non-Tfh CD3+CD4+ T cells are then separated in naive (CD45RA+CCR7+), central memory (CD45RA−CCR7+), effector memory (CD45RA−CCR7−) and terminally differentiated (CD45RA+CCR7−). (TIF) [file ppat.1005287.s003.tif]

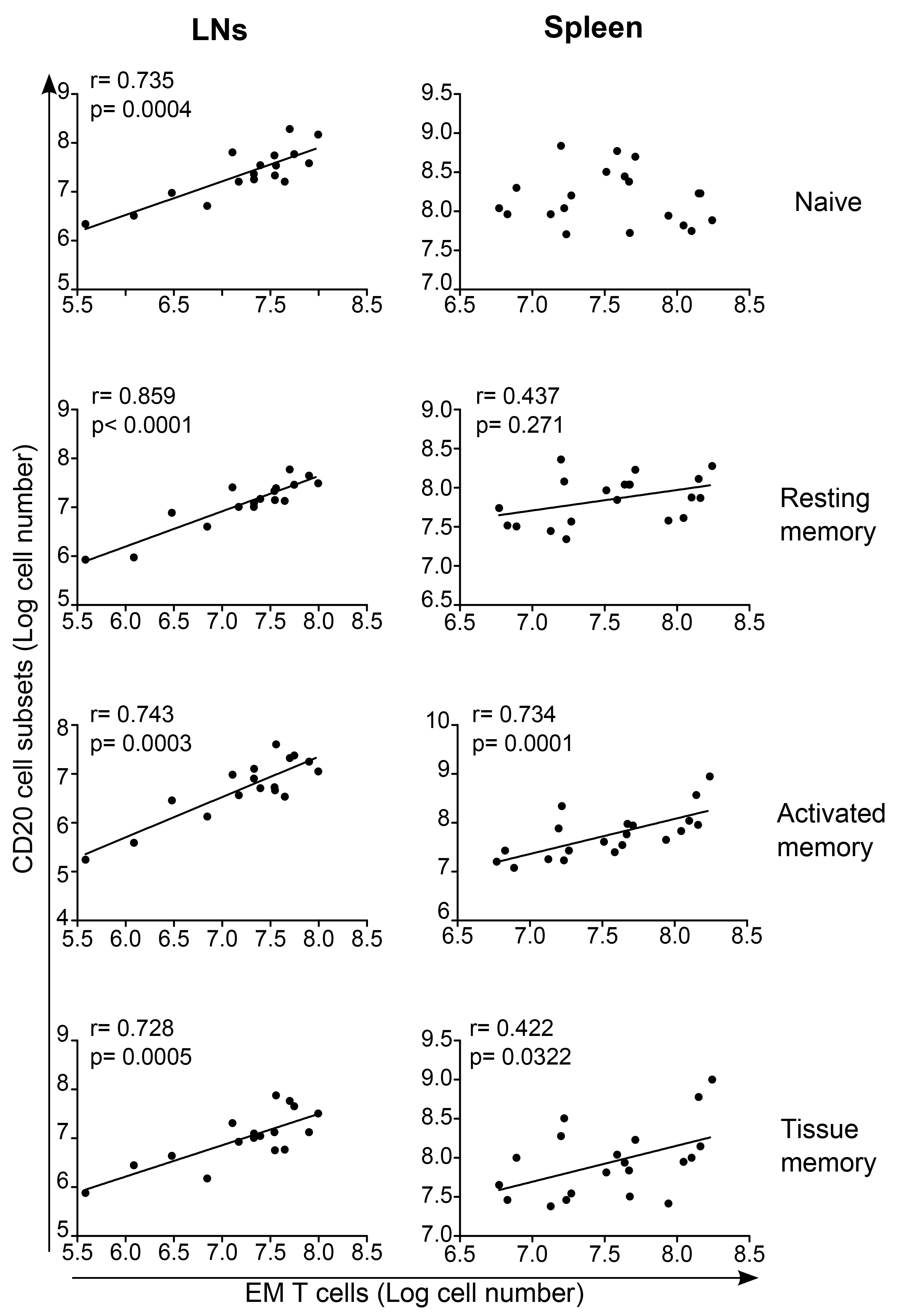

Supplement: S4 Fig — Diagrams show correlation between the percentage of effector memory cells and the percentage of B cell subset (as defined in fig 5) in LNs and spleen of RMs. Each dot represents an individual RM. Spearman analysis was used for correlations. (TIF) [file ppat.1005287.s004.tif]

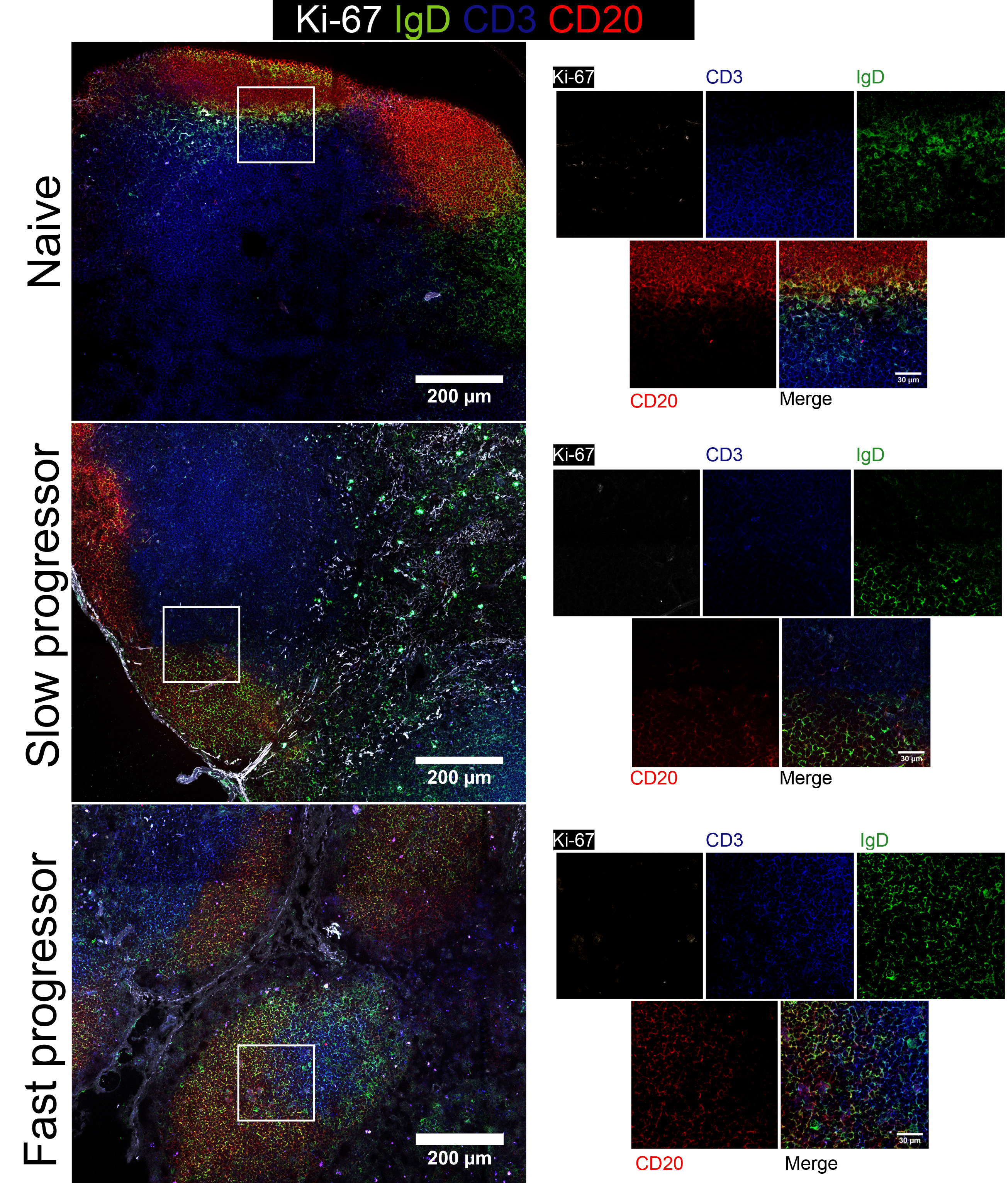

Supplement: S5 Fig — LN tissue sections were stained with antibodies against Ki-67 (white), IgD (green), CD3 (blue) and CD20 (red) and imaged by confocal microscopy. Representative pictures of a naive RM and of two chronically SIV-infected RMs, slow and fast progressor RMs are shown. The picture is representative of two individuals animals performed independently. Higher magnification is shown on the right part of the picture. Scale is shown. (TIF) [file ppat.1005287.s005.tif]

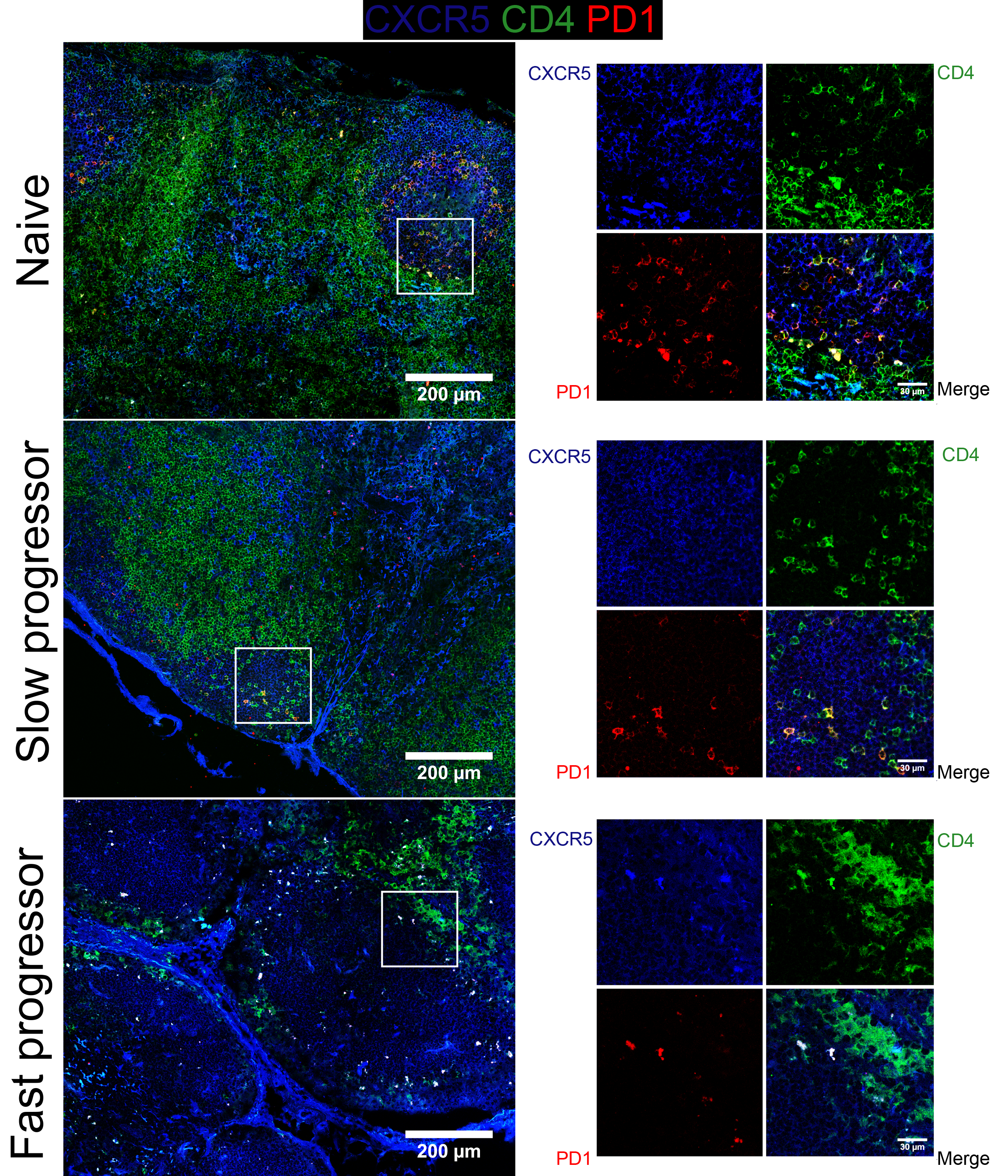

Supplement: S6 Fig — LNs tissue sections were stained with antibodies against CXCR5 (blue), CD4 (green) and PD-1 (red) and imaged by confocal microscopy. Representative pictures of the same animals as depicted in S5 Fig are shown. Higher magnification is shown on the right part of the picture. Scale is shown. (TIF) [file ppat.1005287.s006.tif]

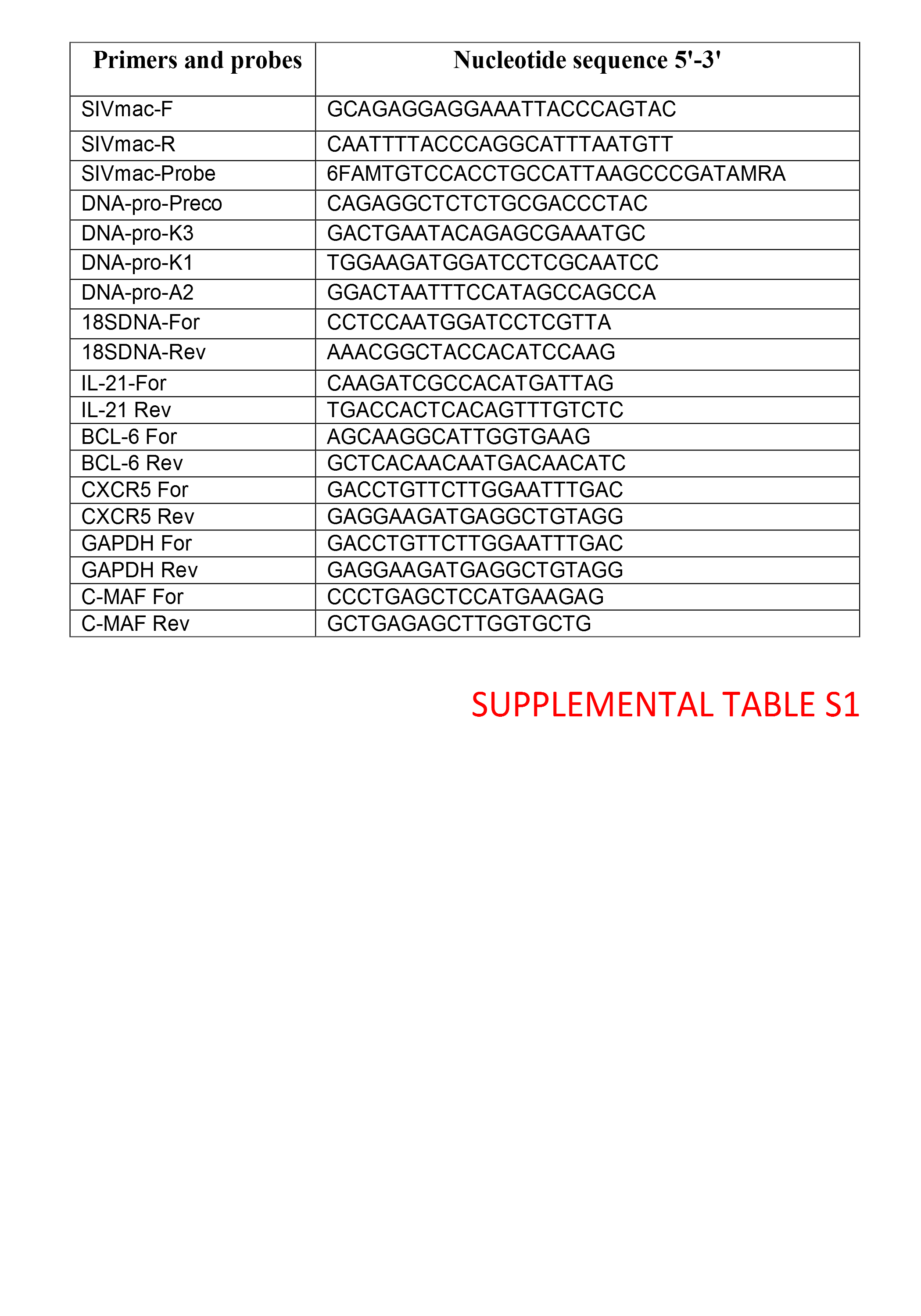

Supplement: S1 Table — (PNG) [file ppat.1005287.s007.png]

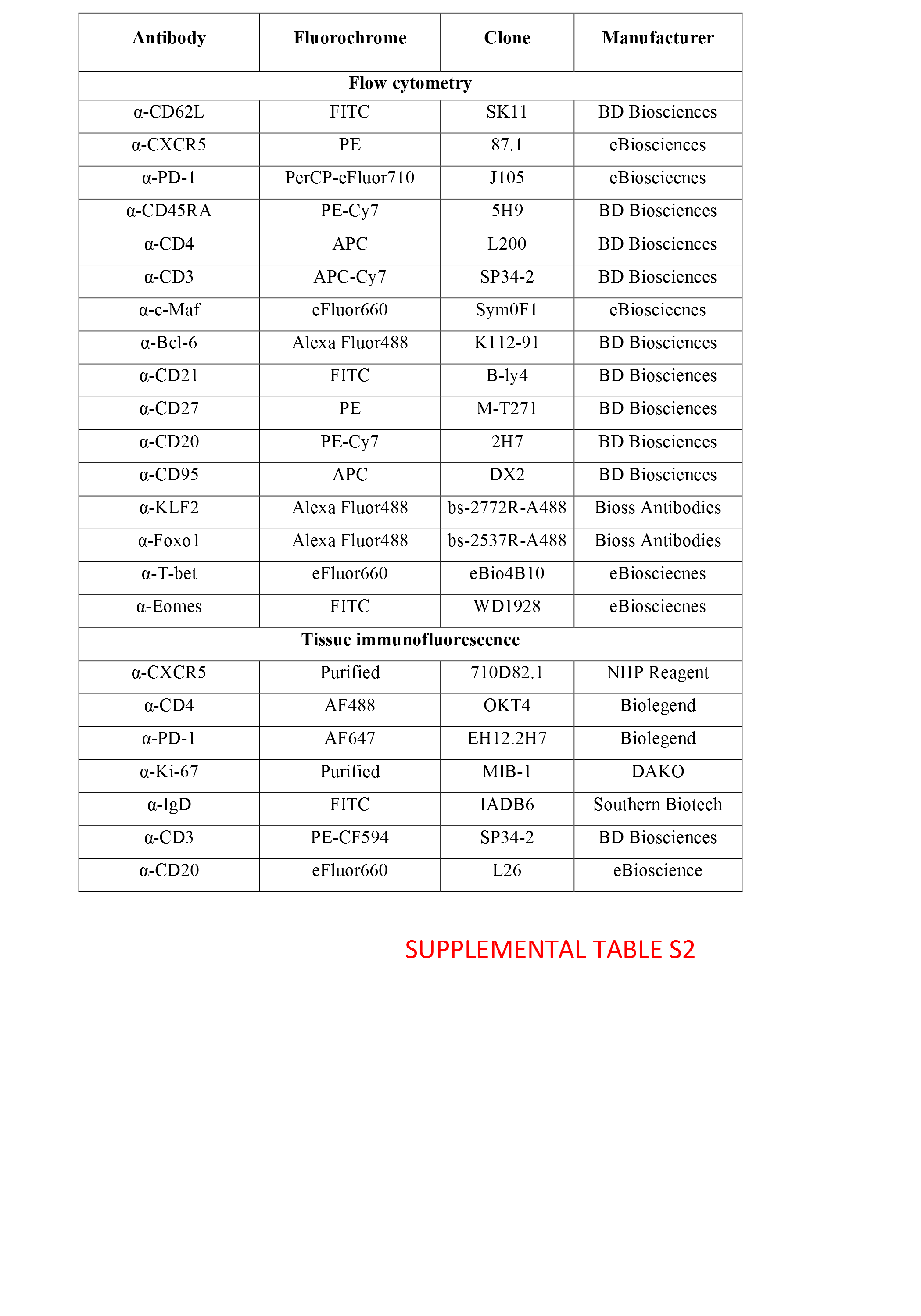

Supplement: S2 Table — Flow cytometry and cell sorting (upper list), tissue immunofluorescence (bottom list). (PNG) [file ppat.1005287.s008.png]
